# Metabolomics reveals the differential regulatory mechanisms of quality and flavonoid biosynthetic pathways during the drying process of varieties licorice

**DOI:** 10.1016/j.fochx.2025.102631

**Published:** 2025-06-04

**Authors:** Lichun Zhu, Mengqin Li, Xuetao Zhang, Qian Zhang, Xuhai Yang, Zhihua Geng

**Affiliations:** a College of Mechanical and Electrical Engineering, Shihezi University, Shihezi, China; b Xinjiang Production and Construction Corps Key Laboratory of Modern Agricultural Machinery, Shihezi, China; c Engineering Research Center for Production Mechanization of Oasis Special Economic Crop, Ministry of Education, Shihezi, China

**Keywords:** Flavonoid, Metabolism, Drying, Antioxidant capacity, Licorice

## Abstract

Flavonoids are one of the major chemical components in licorice. However, during the drying process, their chemical stability, content, and biological activity can all change. Moreover, the impact of different varieties on these aspects is still not clear. This study employed hot air-drying technology at 45–65 °C to examine the effect of drying temperature on the drying flavonoids, total phenolics, and antioxidant activity and other quality aspects in *Glycyrrhiza uralensis* and *Glycyrrhiza inflata* from the Xinjiang Uygur Autonomous Region, China. Results showed that the optimal drying temperature was 60 °C, at which the total phenolic content of both varieties of licorice reached its peak, with *G. inflata* achieving 2.42 mg GAE/g and *G. uralensis* reaching 2.46 mg GAE/g. However, drying significantly reduced the antioxidant activity of licorice. Additionally, there were 24 differential flavonoid metabolites between the dried samples of *G. inflata* and *G. uralensis*, among which 19 were up-regulated and 5 were down-regulated. *G. uralensis* retained better overall quality than *G. inflata* after drying. These findings provide insights into flavonoid metabolite and antioxidant regulation under drying stress, and valuable information for primary processing in licorice-producing areas.

## Highlights

• Drying significantly affected flavonoid content and antioxidant activity in licorice.
• 24 flavonoid metabolites show differential expression in *Glycyrrhiza uralensis* vs *Glycyrrhiza inflata* after drying.
• *Glycyrrhiza uralensis* retained better overall quality than *Glycyrrhiza inflata* after drying.

## Graphical abstract

**Figure:**
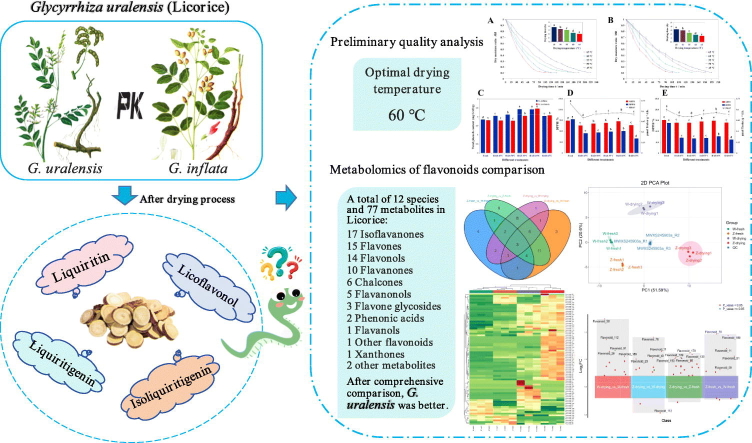

## 1 Introduction

*Glycyrrhiza uralensis* (licorice) is a traditional medicinal herb that is widely used in (Nishidono & Tanaka, 2025) medicines, foods, and cosmetics (Shaikh et al., 2024). Chinese licorice products, such as glycyrrhizic acid monoamine salt, glycyrrhizin, and flavonoid extracts, hold a share of the international market and are primarily exported to Asia, Europe, and North America (Wu et al., 2024). Licorice is rich in flavonoids, which exhibit numerous biological activities, including anti-oxidation, anti-inflammation, and anti-tumor attributes (Yang et al., 2019). Network pharmacology studies (Patil et al., 2021) have shown that licorice flavonoids may act on multiple targets and signaling pathways, including anti-tumor, anti-inflammatory, endocrine regulation, and amino acid metabolism. Thus, flavonoids play an important role in maintaining health and enhancing immunity, and they are popular with consumers. Owing to its unique geographical conditions, Xinjiang has become the largest licorice-producing area in China (Abudurezike et al., 2023), with an abundance of germplasm resources and several licorice varieties. However, fresh licorice is difficult to store and is easily damaged by airborne mildew; to ensure drug safety, if mildew is detected, the entire batch of licorice products is destroyed or reprocessed, which significantly affects production costs (Wei et al., 2013). An appropriate degree of drying can extend the shelf life and prevent mildew deterioration during storage. Moreover, dried licorice is convenient for packaging and transportation, and the lower water content is conducive to reducing the influence of water on efficiency and quality during the extraction process (Zhu et al., 2024), which can reduce the cost of subsequent drying treatments. Therefore, drying is an essential step in licorice processing.

The moisture content of freshly extracted licorice samples must be reduced from 40 %–50 % to the pharmacopoeia-recommended safe level of 10 %–12 % when drying. Long-term heat stress accelerates the metabolism of internal flavonoids, leading to the notable loss of heat-sensitive components (Zhu et al., 2023). Licorice contains important medicinal ingredients, such as liquiritin and liquiritigenin, both of which are flavonoids (Li et al., 2024). However, most research on licorice is focused on its therapeutic applications in later stages currently. There is almost no research on the initial processing of licorice at its place of origin. Shang et al. (2023) have used far-infrared drying for licorice and studied its drying characteristics and quality impact, but this research is not in-depth. It is even less comprehensive when it comes to the detection, identification, and quantification of flavonoids in dried licorice, which provide a variety of physiological functions. Moreover, there are no reports in the literature on which metabolic pathways lead to the changes in flavonoid content in licorice. And it is unclear which of the two main species in the Xinjiang Uygur Autonomous Region, *G. uralensis* Fisch. or *G. inflata* Batalin, has higher medicinal value and quality after drying.

To address this issue, *G. uralensis* and *G. inflata* were dried at different temperatures, and their characteristics were compared. By analyzing the drying kinetic characteristics, basic quality, and antioxidant activities of fresh and hot air-dried licorice, the changes in antioxidant capacity were elucidated, and a preliminary optimal drying temperature was determined. Flavonoid metabolomics analysis was performed on the optimal samples. Metabolomics was used to analyze the flavonoids in licorice before and after drying and to explore the effects of drying treatments on flavonoid content and metabolism. The results provide new insights into the differences between important licorice varieties in the Xinjiang Uygur Autonomous Region and the changes in flavonoid quality under drying stress.

## 2 Materials and methods

### 2.1 Materials

Fresh licorice samples for each variety obtained from the same batch were purchased in Bachu County, Kashgar Prefecture, Xinjiang Uygur Autonomous Region, China (38°46′ N, 78°22′ E, 1116 m above sea level). After fresh excavation, the head and root of each reed were removed, and the main root in the middle was retained. The root strips were refrigerated at 4 °C in plastic wrap until testing. The two licorice varieties were *G. uralensis* and *G. inflata*. Fresh *G. uralensis* root strip is brown in appearance but reddish in color, whereas fresh *G. inflata* is brown in appearance but yellowish in color, and the interior of both varieties is yellowish when cut.

### 2.2 Drying equipment and methodology

Referring to the research methods of Zhu et al. (2023) with slight modifications. To ensure that the raw samples of both licorice varieties were uniform for the drying test, damaged and rotten parts were removed before the test, and the side branches and roots were cut together to preserve the trunk root strip. *G. uralensis* and *G. inflata* samples with uniform licorice root strips (thickness 10 ± 0.5 mm in diameter) and smooth and plump root strips were manually selected for testing. The strips were cleaned of surface soil, sliced (4 ± 0.5 mm), and then laid flat on a drying tray. The total net weight of the licorice on each tray was 100 ± 5 g. The drying temperature variable was set at 45 °C, 50 °C, 55 °C, 60 °C, and 65 °C referring to the research methods of Shang et al. (2023) and making an extension. The drying process was carried out using a standard hot air-drying oven (DHG-9070 A; Power 1550 W; Voltage 220 V; temperature adjustment range RT + 10–250 °C; Shanghai Yiheng Technology Co., Ltd.). The hot air-drying oven was turned on before testing and preheated for 30 min at the set temperature. When the temperature in the drying chamber reached the preset temperature and the equipment was stable, a tray containing flat licorice tablets was inserted for drying. Samples were weighed every 15 min. When the licorice moisture content was <12 % (referring to Pharmacopoeia of the People's Republic of China I, 2020), drying was terminated. The dry material tray was removed, set aside to cool under ambient conditions, sealed with a standard sealed bag, and refrigerated at 4 °C. The tests were conducted in triplicate to ensure data reliability.

### 2.3 Evaluation indicators

#### 2.3.1 Initial moisture content

The initial dry-basis moisture content of licorice was determined using the 105 °C oven method (referring to Pharmacopoeia of the People's Republic of China I, 2020). When the licorice mass change was ≤2 mg before and after drying at a constant temperature of 105 °C, it was considered to have reached a constant weight, and the moisture content was recorded as the initial moisture content of licorice using Eq. (1):

(1) $$M_{0}=\frac{W_{0}-W_{d}}{W_{0}}\times 100\%$$

where *M*_*0*_ is the initial moisture content of the licorice (%), *W*_*0*_ is the initial mass of the licorice (g), and *W*_*d*_ is the absolute dry weight (g).

#### 2.3.2 Moisture ratio

The dry-basis moisture content of the licorice at time *t* (Hou et al., 2024) was calculated using Eq. (2):

(2) $$M_{t}=\frac{W_{t}-W_{d}}{W_{d}}$$

where *M*_*t*_ is the dry basis moisture content (g/g), *t* is the drying time (min), *W*_*t*_ is the total mass of material at drying time *t* (g), and *W*_*d*_ is the absolute dry mass of the licorice (g).

The equilibrium dry basis water content, *M*_*e*_, was considerably smaller than *M*_*0*_ and *M*_*t*_; therefore, Eq. (2) was reduced to Eq. (3):

(3) $$\mathrm{MR}=\frac{M_{t}}{M_{0}}$$

The drying rate of licorice during the drying process (Huang, Wang, et al., 2025) was obtained using Eq. (4):

(4) $$\mathrm{DR}=\frac{M_{t1}-M_{t2}}{t_{1}-t_{2}}$$

#### 2.3.3 Total phenols

The total phenol content of the licorice samples was determined using ultraviolet spectrophotometry. The Folin-Ciocalteu method (referred to as the FC method) was modified according to a previous study (Geng et al., 2023), and a UV-1900i ultraviolet spectrophotometer (Shimazu, Shanghai Experimental Equipment Co., Ltd., Shanghai, China) was used to determine the total phenol content. This device was used for the subsequent determination of antioxidant capacity. Before the determination, the ultraviolet spectrophotometer was preheated for 30 min.

Licorice samples were ground, passed through a 60-mesh sieve, and then set aside. The polished powder of a licorice sample (0.03 g) was collected, and 1.5 mL of 60 % ethanol was added to prepare the test solution. An L6–180 ultrasonic cleaning machine (Shanghai Haozhuang Instrument Co., Ltd., Shanghai, China) was used for sample extraction by oscillation at 60 °C for 2 h (if there is evaporation during the process, the volume is fixed with 60 % ethanol to 1.5 mL). Samples were then centrifuged at 24 ± 2 °C and 12,000 rpm for 10 min, 40 μL of the supernatant was extracted, and the corresponding reagents in the total phenol content determination kit (G0117F, Suzhou Gris Biotechnology Co., Ltd.) were added based on the Folin phenol method according to the manufacturer's instructions. Samples were then mixed and subjected to a light-shielding reaction at 24 ± 2 °C. After a full reaction for 1 h, the light absorption at 760 nm was determined. The standard curve was drawn using gallic acid as the standard substance, and the total phenol content of the sample was expressed as GAE (the proportional value of gallic acid). Samples were measured in triplicate in each group, and the average was recorded.

#### 2.3.4 Antioxidant capacity

The antioxidant activity of the supernatant was determined according to the scavenging capacity of DPPH (2, 2-diphenyl-1-picryl-hyacyl), ABTS+ (2, 2-azinobis (3-ethylbenzothiazole-6-sulfonic acid), and FRAP (iron-reducing antioxidant capacity). The sample extract solution (80 % methanol) was mixed with the sample in an ice bath homogenate (1 g of licorice powder with 10 mL of extract solution) at a ratio of 1:10 using a DPPH Free Radical Scavenging kit (G0128F, Suzhou Gris Biotech Co., Ltd.) according to the manufacturer's instructions. After full extraction, the supernatant was obtained by centrifugation at 4 °C and 10,530 rpm for 10 min. The standard solution provided in the kit was prepared according to the instructions, the absorbance was measured at the specified wavelength (517 nm), and a standard curve was plotted. The sample and reaction solutions were mixed, and the reaction was conducted using a UV-1900i ultraviolet spectrophotometer according to the manufacturer's instructions. The absorbance was determined, and the antioxidant capacity of the sample was calculated using the standard curve. The experiment was conducted in triplicate for each sample, and the standard deviation (SD) and coefficient of variation (CV <5 %) were calculated to evaluate the repeatability and stability of the data. An ABTS free radical scavenging capacity kit (G0127F, Suzhou Gris Biotechnology Co., Ltd., Suzhou, China) and FRAP Total antioxidant capacity kit (G0115W, Suzhou Gris Biotechnology Co., Ltd., Suzhou, China) were used in accordance with the manufacturer's instructions.

### 2.4 Flavonoid metabolomics

#### 2.4.1 Sample preparation and extraction

Licorice samples from each of the two varieties (including the skin of the licorice tablets) were ground to a powder (20 g) with liquid nitrogen, and then extraction liquid (10 μL) was added to each powder sample. The extraction liquid was prepared using 500 μL of 70 % methanol solution from the internal standard mixed working liquid at a concentration of 4000 nmol/L. After ultrasonic extraction for 30 min, the supernatant was centrifuged at 4 °C and 12,000 rpm for 5 min, and the sample was filtered through a 0.22 μm filter membrane and stored in a sample vial for follow-up analysis.

#### 2.4.2 Reagents and instruments

Liquid chromatography-tandem mass spectrometry, also called LC-MS/MS (QTRAP 6500+, SCIEX, Shanghai, China) was used for the flavonoid metabolomics determination. The main reagents were HPLC-grade methanol (MeOH; Merck, Darmstadt, Germany), formic acid (Sigma-Aldrich, St Louis, MO, USA), and hydrochloric acid (Xinyang Chemical Reagent, China). Standard solutions (purity >98 %) were purchased from MCE (Shanghai, China). The stock solutions of the standards were prepared at a concentration of 10 mmol/L in 70 % MeOH.

#### 2.4.3 Acquisition conditions of chromatography–mass spectrometry

Utilizing an UPLC-ESI-MS/MS system, the licorice sample extracts were analyzed. The data acquisition instrument system comprised ultra-high performance liquid chromatography (UPLC, ExionLC™ AD, https://sciex.com.cn/) and tandem mass spectrometry (MS/MS; QTRAP® 6500+, https://sciex.com.cn/). The liquid phase conditions during detection were as follows: chromatographic column, Waters ACQUITY UPLC HSS T3 C18 column (1.8 μm, 100 mm × 2.1 mm i.d.); mobile phase A, ultrapure water (addition of 0.05 % formic acid); mobile phase B, acetonitrile (addition of 0.05 % formic acid); flow rate, 0.35 mL/min; column temperature, 40 °C; injection volume, 2 μL; and elution gradient, 0 min at A/B 90:10 (*V*/V), 1 min at A/B 80:20 (V/V), 9 min at 30:70 (V/V), 12.5 min at A/B 5:95 (V/V), 13.5 min at 5:95 (V/V), 13.6 min at 90:10 (V/V), and 15 min at 90:10 (V/V).

Linear ion trap (LIT) and triple quadrupole (QQQ) scans were acquired on a triple quadrupole-linear ion trap mass spectrometer (QTRAP), QTRAP® 6500+ LC-MS/MS System, equipped with an ESI Turbo Ion-Spray interface, operating in positive and negative ion mode and controlled by Analyst 1.6.3 software (Sciex). The mass spectra conditions for the detection of flavonoids in licorice were as follows: electrospray ionization (ESI) source temperature, 550 °C; mass spectrum voltage, 5500 V in positive ion mode and − 4500 V in negative ion mode; and curtain gas (CUR), 35 psi. In Q-Trap 6500+, each ion pair was scanned and detected according to the optimized declustering potential (DP) and collision energy (CE).

#### 2.4.4 Data analysis

The mass spectrum data was processed using Analyst 1.6.3 software, Fig. S1 and S2 shows the total ion flow diagram (TIC) and extracted ion flow diagram (XIC). MultiQuant 3.0.3 software were used to process the mass spectrum data, Fig. S3 shows the quantitative analysis and integral correction results of a random substance in different samples. Both show the stability of the instrument and the reliability of the data. The TIC chart of quality control sample (Fig. S4) and coefficient of variation (CV) distribution in each group of samples (Fig. S5) further illustrate this point. The standard curves of different substances are drawn and shown in Table S1. The substances detected results of this project are shown in Table S2. Above chart information detailed in Supplementary Information A.

In addition, principal component analysis (PCA) was performed on samples (including quality control samples) to preliminarily determine the overall metabolic differences among samples of each group and the degree of variation among samples within the group. Subsequently, samples were analyzed using cluster analysis, orthogonal partial least squares discriminant analysis (OPLS-DA), differential metabolite screening, Kyoto Encyclopedia of Genes and Genomes (KEGG) function annotation, and enrichment analysis.

## 3 Results

### 3.1 Drying quality of *G. uralensis* and *G. inflata*

#### 3.1.1 Drying kinetic curve

**Fig. 1:**
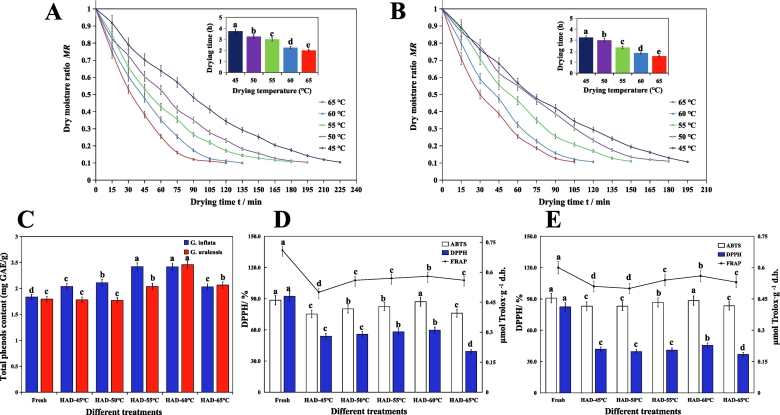
Determination of basic quality and antioxidant capacity of licorice samples under different treatment methods. A: Drying kinetics curve and drying time of *Glycyrrhiza uralensis.* B: Drying kinetics curve and drying time of *Glycyrrhiza inflata.* C: Total phenol content in licorice samples under different treatment methods of *G. uralensis* and *G. inflata.* D: Determination of antioxidant capacity of *G. uralensis* using the DPPH, ABTS, and FRAP methods*.* E: Determination of antioxidant capacity of *G. inflata* using the DPPH, ABTS, and FRAP methods*.*

According to the weights determined at 15-min intervals, drying water ratio curves of *G. uralensis* and *G. inflata* at 45 °C, 50 °C, 55 °C, 60 °C, and 65 °C were plotted. The drying time decreased with increasing temperature (Fig. 1A and B). The shortest drying times for both varieties occurred at 65 °C (up to 1.75 h), and the longest drying times occurred at 45 °C (up to 3.75 h) [*P* < 0.05]. Moreover, *G. inflata* dried slightly faster than *G. uralensis* at each drying temperature, which may reflect its lower initial moisture content. The drying rates of *G. inflata* and *G. uralensis* showed a general decreasing trend, which became more obvious with increasing temperature. At 45 °C and 50 °C, the drying trends of *G. inflata* were similar, whereas *G. uralensis* showed obvious differences caused by the temperature gradient.

#### 3.1.2 Total phenolic content

Ultraviolet (UV) spectrophotometry was used to measure the total phenolic content of the two licorice varieties before and after drying. There were no significant differences in the fresh samples; however, the phenolic levels of *G. inflata* were slightly higher than those of *G. uralensis*. For both species, the contents of the dried samples differed from those of the fresh samples. The highest total phenolic content for both species occurred at 60 °C drying, with the content of *G. uralensis* slightly higher than that of *G. inflata*.

The total phenolic content of the dried *G. inflata* samples was higher than that of the fresh samples (Fig. 1C). The total phenolic contents were similar at drying temperatures of 45 °C, 50 °C, and 65 °C and almost the same at drying temperatures of 55 °C and 60 °C. However, at a drying temperature of 60 °C, the total phenol content in the dried samples peaked (up to 2.42 mg GAE/g).

*Glycyrrhiza uralensis* and *G. inflata* showed different trends in total phenolic content after drying. In *G. uralensis*, the total phenolic content of the dried samples varied, appearing both higher and lower than that of the fresh samples. The highest total phenolic content was 2.46 mg GAE/g at 60 °C, whereas the total phenolic content of the dried samples was lower than that of the fresh samples at 45 °C. The lowest total phenolic content, 1.77 mg GAE/g, was achieved at 50 °C, and the total phenolic content in the dried samples was similar at 55 °C and 65 °C.

#### 3.1.3 Antioxidant capacity

The DPPH, ABTS, and FRAP methods were used to evaluate the antioxidant activities of the fresh and dried licorice samples under different temperature conditions (Fig. 1D and E). For both varieties and all three methods, the antioxidant activities of the dried samples were lower than those of the fresh samples.

The DPPH clearance rate of fresh *G. inflata* was 82.35 %, whereas that of dried *G. inflata* decreased under different drying temperatures, with the lowest observed at 65 °C (36.68 %) and the highest observed at 60 °C (45.52 %). The DPPH clearance rate of fresh *G. uralensis* was 92.35 %; that of dried *G. uralensis* also showed a downward trend under different drying temperatures, with the lowest observed at 65 °C (39.13 %) and the highest observed at 60 °C (59.73 %). Fresh and dried *G. uralensis* samples under the same drying conditions showed higher DPPH clearance values than the fresh *G. inflata* samples.

The ABTS value of fresh *G. inflata* was 90.71 %; with the lowest observed at 45 °C (82.99 %) and the highest observed at 60 °C (88.42 %) for dried samples. Similarly, *G. uralensis* exhibited the worst performance at 45 °C (82.99 %) and the best performance at 60 °C (86.99 %).

The FRAP values of *G. inflata* and *G. uralensis* were 0.60 and 0.71 μmol Trolox/g d.m, respectively. The optimal temperature for both species was 60 °C, with *G. inflata* reaching 0.56 μmol Trolox/g d.m and *G. uralensis* reaching 0.58 μmol Trolox/g d.m.

### 3.2 Metabolomics of flavonoids

#### 3.2.1 Quantitative and categorical analysis of flavonoid metabolites

**Fig. 2:**
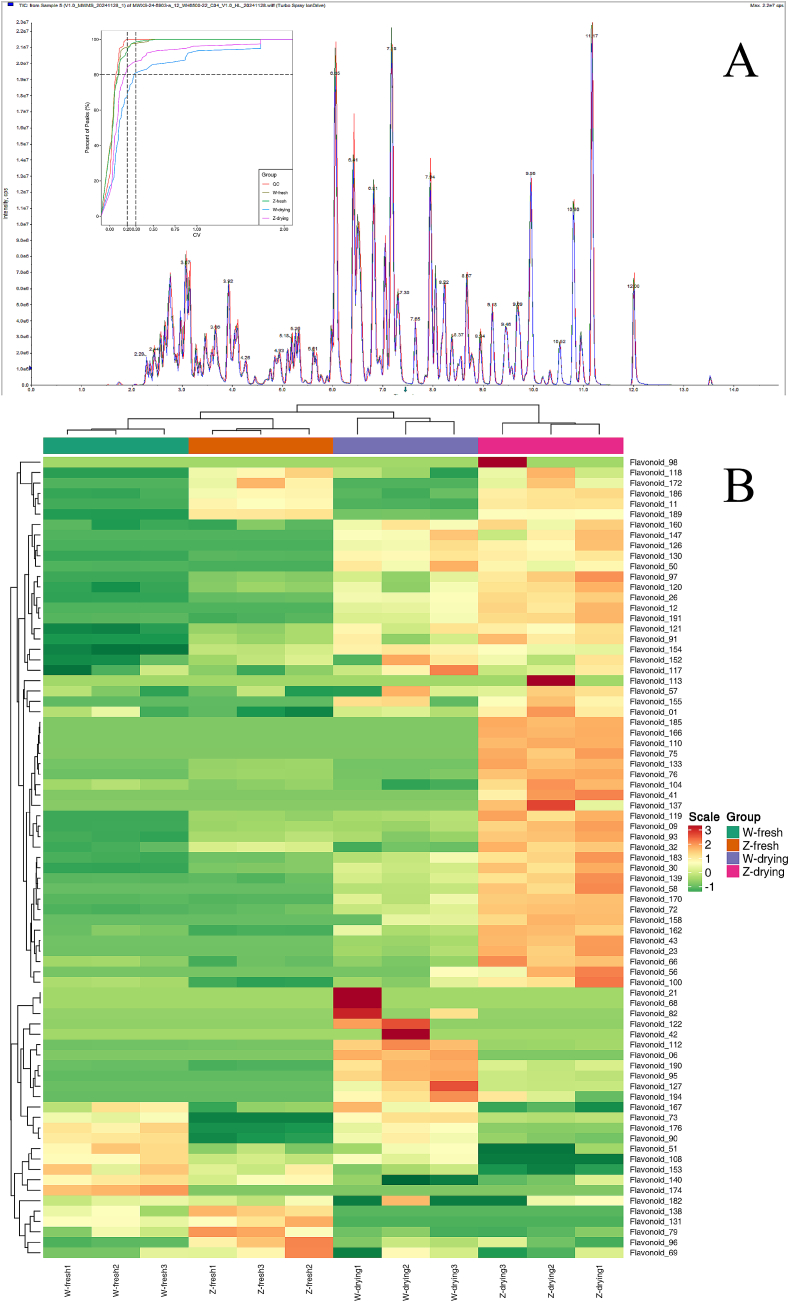
A: Total ion current map of metabolite detection of licorice samples. B: Clustering analysis diagram of licorice samples. (W-Fresh: Fresh sample of *G. uralensis*; *Z*-Fresh: Fresh sample of *G. inflata*; W-Drying: Dried sample of *G. uralensis*; Z-Drying: Dried sample of *G. inflata*.)

The total ion current map of metabolite detection is presented in Fig. 2A. The total ion current curves of metabolite detection exhibited high overlap; that is, the retention times and peak intensities were consistent, indicating that the signal stability of each sample was good when detected at different times using mass spectrometry (Xin et al., 2025). Moreover, according to the CV distribution, the proportion of substances with CV values <0.2 in QC samples was >80 %, indicating that the licorice test data were highly stable.

In total, 12 species and 77 metabolites were detected in all licorice samples (Fig. 2B), including 17 isoflavanones, 15 flavones, 14 flavonols, 10 flavanones, 6 chalcones, 5 flavanonols, 14 flavonols, 3 flavone glycosides, 2 phenonic acids, 1 flavanol, 1 other flavonoid, 1 xanthone, and 2 other metabolites. A detailed list of substances can be found in Supplementary A-Table S2.

A total of 48 flavonoid compounds were identified in fresh *G. inflata* samples; the top three flavonoid compounds were: liquiritin (755.38 ± 26.55 nmol/g), naringenin-7-glucoside (121.09 ± 1.60 nmol/g), and licoisoflavone a (90.62 ± 2.52 nmol/g). Conversely, 67 flavonoid compounds were detected in dried samples; the top three were liquiritin (702.37 ± 24.29 nmol/g), licoflavonol (340.17 ± 8.37 nmol/g), and naringenin-7-glucoside (164.44 ± 0.63 nmol/g). Compared with fresh samples, 19 additional substances were identified in dried *G. inflata* samples. In contrast, some substances were no longer detected after drying.

A total of 47 flavonoid compounds were identified in the fresh *G. uralensis* samples; the top three were liquiritin (712.93 ± 17.88 nmol/g), naringenin-7-glucoside (247.61 ± 10.93 nmol/g), and licoisoflavone a (68.71 ± 3.44 nmol/g). In contrast, 64 flavonoid compounds were identified in the dried samples; the top three substances were liquiritin (716.55 ± 37.61 nmol/g), naringenin-7-glucoside (223.99 ± 12.77 nmol/g), and licoflavonol (183.69 ± 40.58 nmol/g). Compared with the fresh samples, the dried *G. uralensis* samples contained 17 additional substances. In contrast, some substances were no longer detected after drying.

#### 3.2.2 Flavonoid differential metabolites

**Fig. 3:**
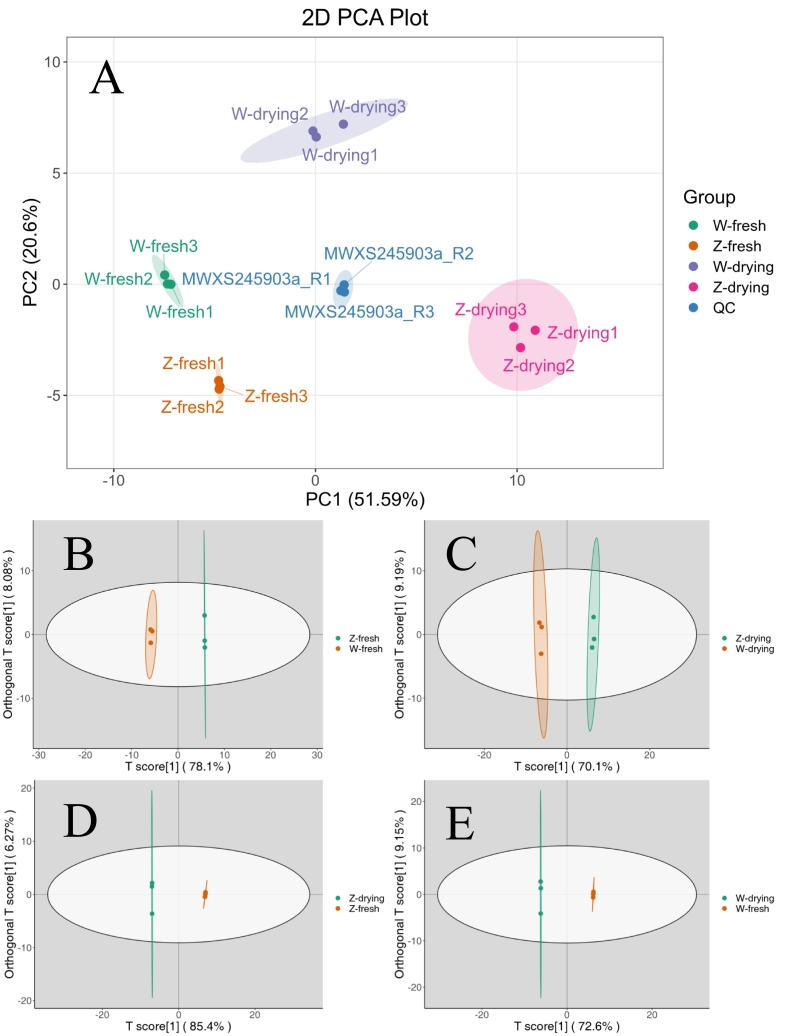
Principal component analysis and orthogonality partial least squares-discriminant analysis of licorice group samples. A: PCA score map of quality spectrum data for licorice group samples*.* B–E: Orthogonality partial least squares–discriminant analysis of licorice group samples*.*

Principal component analysis (PCA) was used to preliminarily understand the overall metabolic differences among the samples in each group and the degree of variation among samples within the group. PC1 and PC2 were 51.59 % and 20.60 %, respectively (Fig. 3A); the *G. inflata* fresh samples, *G. inflata* dried samples, *G. uralensis* fresh samples, and *G. uralensis* dried samples were all far apart, indicating that the metabolomes of the two species differed significantly (Huang, Wang, et al., 2025), and drying significantly affected flavonoid metabolism.

The metabolome data were further analyzed using the orthogonality partial least squares–discriminant analysis (OPLS–DA) model, and the score map CDEF of each group was drawn to show differences between each group. The T-score of fresh *G. inflata* and *G. uralensis* (Fig. 3B) was 78.10 % and the orthogonal T-score was 8.07 %. For dried *G. inflata* and *G. uralensis* (Fig. 3C), the T-score was 70.30 %, and the orthogonal T-score was 9.22 %. The T-score of *G. inflata* before and after drying (Fig. 3D) was 85.70 %, and the orthogonal T-score was 6.10 %. For the *G. uralensis* samples before and after drying (Fig. 3E), the T-score was 72.60 % and the orthogonal T-score was 9.19 %. In summary, different varieties at different stages were effectively distinguished.

The fresh samples of *G. inflata* and *G. uralensis* contained 18 differential metabolites, including 12 up-regulated and 6 down-regulated metabolites. We identified 24 differential metabolites between dried *G. inflata* and *G. uralensis* samples, of which 19 were up-regulated and 5 were down-regulated. In the *G. inflata* samples, 41 differential metabolites were identified before and after drying, including 37 up-regulated and 4 down-regulated metabolites. In *G. uralensis* samples, 30 differential metabolites were observed, with 27 up-regulated and 3 down-regulated metabolites.

**Fig. 4:**
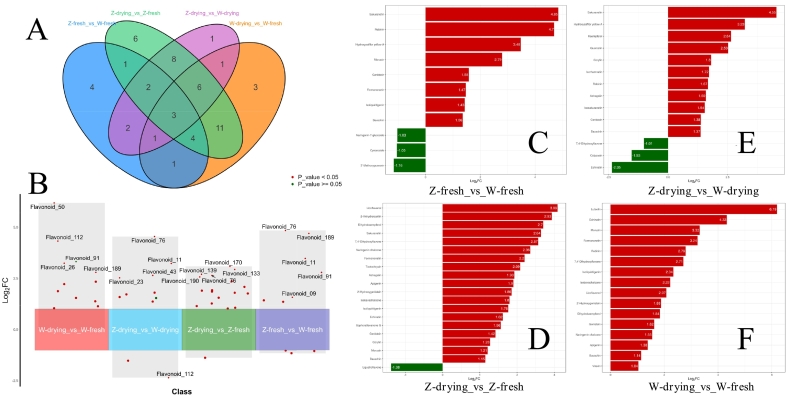
A: Venn diagram of differences among groups of licorice samples*.* B: Volcanic map of different substances among groups of licorice samples. C–F: Difference multiples bar chart of licorice group samples*.*

The quantitative metabolite data were compared among all the samples based on the significant fold changes shown in Fig. 4A and B (metabolites with fold changes ≥2 and fold change ≤0.5 were selected; if the difference in metabolites was more than twice or less than half, the difference was considered to be significant (Huang & Xu, 2024)). Between fresh samples of *G. inflata* and *G. uralensis*, the three most up-regulated substances were sakuranetin, robinin, and hydroxysafflor yellow A (Fig. 4C). Metabolites with significant changes in *G. inflata* samples before and after drying are shown in Fig. 4D, among which the top three up-regulated substances were licoflavonol, β-anhydroicaritin, and dihydrokaempferol. Metabolites with significant changes in *G. inflata* and *G. uralensis* after drying are shown in Fig. 4E, among which sakuranetin, hydroxysafflor yellow A, and kaempferol were the top three up-regulated substances. Metabolites with significant changes in *G. uralensis* samples before and after drying are shown in Fig. 4F, among which luteolin, echinatin, and morusin were the top three up-regulated substances.

In summary, the results showed significant differences in the number and fold changes of up-regulated and down-regulated differential metabolites among groups, suggesting that different licorice varieties and drying treatments have significant effects on the production of differential metabolites.

#### 3.2.3 Differential analysis of metabolic pathways

**Fig. 5:**
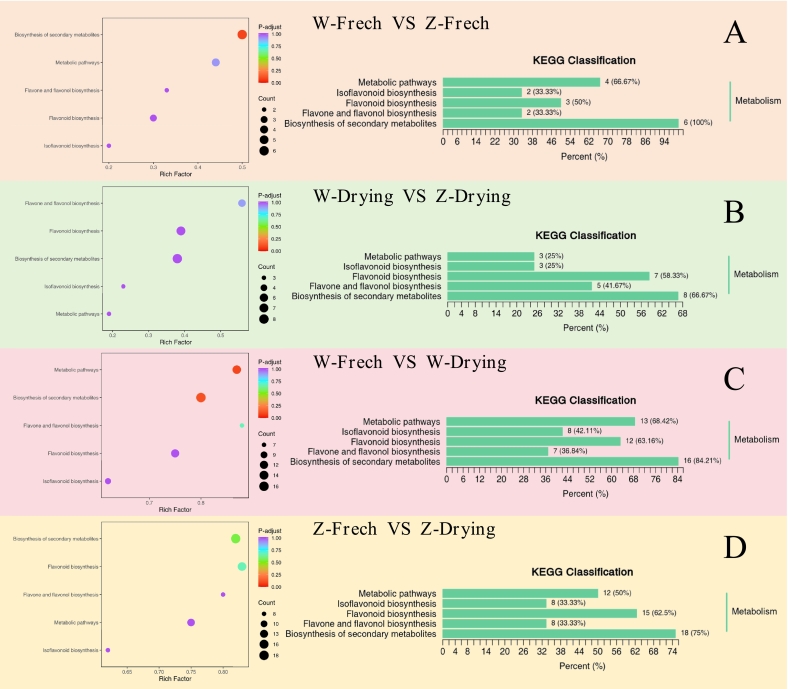
KEGG classification diagram and enrichment analysis diagram of differential metabolites in licorice group samples.

The Kyoto Encyclopedia of Genes and Genomes (KEGG) function annotation of the significantly different metabolites of licorice (Fig. 5) revealed five related signaling pathways: isoflavonoid biosynthesis, metabolic pathways, biosynthesis of secondary metabolites, flavonoid biosynthesis, and flavone and flavonol biosynthesis, which are related to the synthesis of quercetin, kaempferitrin, genistein, and other substances.

In fresh *G. inflata* and *G. uralensis*, pathways were predominantly related to the biosynthesis of secondary metabolites (Fig. 5A), specifically the up-regulation of formononetin, genistein, isoliquiritigenin, sakuranetin, and astragalin and the down-regulation of luteolin.

In dried *G. inflata* and *G. uralensis*, pathways were predominantly related to the up-regulation of astragalin and 3,7-di-*O*-methylquercetin and the down-regulation of calycosin in the biosynthesis of secondary metabolites (Fig. 5B). This was followed by the flavonoid biosynthesis pathway, which enhanced the synthesis of flavonoids and up-regulated the expression of hesperetin, isosakuranetin, and sakuranetin.

In addition, *G. inflata* was predominantly associated with the biosynthesis of secondary metabolites, flavonoid biosynthesis, and metabolic pathways (Fig. 5C); similar results were found for *G. uralensis* before and after drying (Fig. 5D).

## 4 Discussion

Licorice is a high-value medicinal plant with wide application prospects owing to its excellent anti-inflammatory, anti-cancer, and anti-oxidation activities, along with other effects and rich bioactive components. Flavonoids are the main active components isolated from the roots and rhizomes of licorice. A variety of flavonoids and > 300 flavonoid monomer components have been identified in licorice in the past 10 years (Huang, Zhang, et al., 2025), including dihydroflavonoids, chalcones, and isoflavonoids. Flavonoids enhance the antioxidant capacity of animals and scavenge free radicals; specifically, the hydrogen atoms on the phenolic hydroxyl groups can combine with peroxyl free radicals to form flavonoid radicals. These flavonoid radicals react with other free radicals to terminate the free radical chain reaction, thereby reducing oxidative stress (Xue et al., 2025). Other studies have shown that the antioxidant capacity of materials after drying is related to the loss and degradation of phenolic and flavonoid substances, such as those in pequi (Delalibera et al., 2024). Metabolomics can also be used to compare specific changes in flavonoid substances and their effects on antioxidant capacity before and after the drying of different types of licorice.

**Fig. 6:**
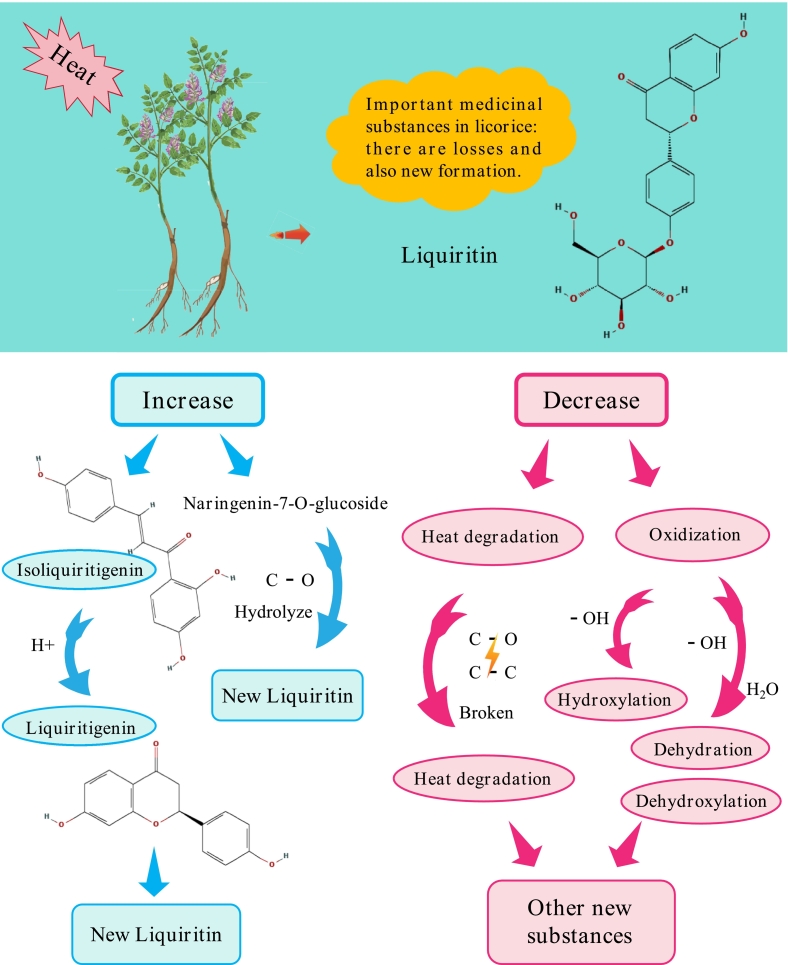
Schematic diagram of the change mechanism of liquiritin.

Liquiritin (CAS: 551–15-5) plays a significant role among the flavonoid components of all Glycyrrhiza species (Zhang, Wang, et al., 2025), and our data revealed that its content ranks first before and after drying. The liquiritin content of *G. inflata* decreased by 7.72 % after drying, whereas that of *G. uralensis* slightly increased by 6.27 %. This indicates that during the licorice drying process, liquiritin is lost, and new liquiritin-related compounds are formed. Schematic diagram of the change mechanism is shown in Fig. 6. This change may occur because the naringenin-7-glucoside (CAS: 529–55-5) content in fresh samples of *G. uralensis* is higher than that in *G. inflata*. Yu et al. (2024) reported that naringenin-7-glucoside can be converted into naringenin (CAS: 529–55-5) through enzymatic action. Moreover, Zuo et al. (2025) demonstrated that naringenin is typically synthesized from naringenin-7-glucoside (naringenin-7-O-glucoside) through enzymatic reactions, a process involving the action of glycosidases, such as β-glucosidase found in licorice (Zuo et al., 2025). Naringenin is a direct precursor of liquiritin (Zhang et al., 2022). Meanwhile, regardless of its status before or after drying, naringenin-7-glucoside ranked second among all flavonoids in licorice. After drying, the naringenin-7-glucoside content in *G. inflata* decreased from 247.61 ± 10.93 nmol/g in fresh samples to 223.99 ± 12.78 nmol/g in dried samples and increased from 121.09 ± 1.60 nmol/g in fresh samples to 164.44 ± 0.63 nmol/g in dried *G. inflata*. Our results are consistent with those of previous studies. Moreover, naringenin-7-glucoside undergoes glucosidic bond hydrolysis on its own under heating conditions (Li et al., 2022), which increases the liquiritin content to a certain extent.

In addition to liquiritin, liquiritigenin (CAS: 578–86-9) and isoliquiritigenin (CAS: 961–29-5), which play major roles in the pharmacological effects of flavonoids (Qin et al., 2022), were also identified. Liquiritigenin is a flavanone that exhibits extensive pharmacological activities. The chemical structure of the compound comprises benzene, furan, and jasmine-like rings (Jiang et al., 2020), and its molecular formula is C_15_H_12_O_4_. During the detection of flavonoids in fresh samples, liquiritigenin was not detected. However, after drying, both varieties of licorice produced new substances, among which liquiritigenin ranked first in terms of content. Hu et al. (2019) also reported the presence of liquiritigenin in fresh licorice, albeit at lower concentrations. The undetectable content may be because the liquiritigenin levels in the fresh samples were less than the detection method's minimum limit. In addition, the high moisture content of fresh licorice may dilute the concentration of liquiritigenin, making it difficult to detect. The disruption of the cell structure during drying may lead to the breakage of covalent bonds between cell walls and intracellular substances, facilitating the release of liquiritigenin from cells and thereby increasing its extraction rate. At the same time, it is possible that the drying process promotes the biosynthesis of Liquiritigenin. As shown in the study by Muhammad et al. (2020) the Liquiritigenin biosynthetic pathway comprises 6 steps that start from aromatic amino acid metabolism (L-tyrosine and *L*-phenylalanine) and end at Liquiritigenin production. *L*-phenylalanine is converted to cinnamic acid by phenylalanine ammonia-lyase (PAL) and can further be transformed into intermediate products such as p-coumaric acid (Zhang, Xu, et al., 2025). These intermediate products are involved in the synthesis of flavonoids, phenolic acids, and other secondary metabolites. The study by Deng et al. (2025) also mentioned that Liquiritigenin biosynthesis begins with the coupling of p-coumaroyl-CoA and malonyl-CoA. This further indicates that Liquiritigenin is very likely to be synthesized during the drying process. Liquiritigenin and isoliquiritigenin are isomers of each other (Zhang et al., 2021); however, unlike liquiritigenin, isoliquiritigenin is a chalcone. The isoliquiritigenin content detected in the samples of *G. inflata* and *G. uralensis* increased after drying. This may be because other chemical components of Glycyrrhiza species can be transformed into isoliquiritigenin via chemical reactions. Guo et al. (2008) found that flavonoids can undergo rearrangement or transformation during heat treatment. For example, naringenin chalcone can be reduced to isoliquiritigenin under specific conditions. Moreover, Mohamed et al. (2025) found that in the flavonoid biosynthesis pathway of Glycyrrhiza, certain intermediates (such as 4-caffeoyl-CoA) may also undergo non-biological catalytic reactions to generate isoliquiritigenin during heat treatment. These conclusions also confirmed the increased isoliquiritigenin content observed in this study.

In addition, the licoflavonol (CAS: 60197–60-6) content in fresh *G. inflata* samples was 40.02 ± 1.14 nmol/g, increasing to 340.17 ± 11.87 nmol/g after drying. This change ranked first among the increased substances. As an important flavonol in licorice, it has a certain impact on its antioxidant activity. Guo et al. (2016) found that licoflavonol can inhibit the secretion of NO by RAW264.7 macrophages induced by lipopolysaccharide, exerting a better anti-inflammatory effect. In a study on flavonoid accumulation in Amomum tsao-ko fruit, Fu et al. (2025) found that the levels of flavonols in each variety of dried fruits increased after drying, which is consistent with our experimental results. Xue et al. (2025) also found that the flavonol content of green tea significantly increased during the drying process. This was further confirmed by the results of this study, which may be because the drying process removes water from fresh samples, thereby increasing the relative content of licoflavonol. Meanwhile, based on the comparison of fresh and dried *G. uralensis* samples, the licoflavonol content also showed the same trend, with a certain degree of increase. However, the degree of increase was not as high as that of *G. inflata*.

## 5 Conclusion

Metabolomics is a powerful tool for analyzing changes in the levels of metabolic substances. This study compared the differences in flavonoid metabolites between fresh and dried licorice samples under optimal drying conditions (60 °C). We identified 12 species and 77 metabolites, including 17 isoflavanones, 15 flavones, 14 flavonols, 10 flavanones, 6 chalcones, 5 flavanonols, 3 flavone glycosides, 2 phenonic acids, 1 flavanol, 1 other flavonoid, 1 xanthone, and 2 other metabolites. In total, 18 differential metabolites were identified between the fresh samples of the two licorice varieties, and 24 differential metabolites were identified between the dried samples of the two licorice varieties. Changes in the flavonoid metabolites between the two licorice varieties were determined, and their antioxidant capacities were measured. The antioxidant activity of both cultivars decreased after drying, with *G. uralensis* showing greater antioxidant activity than *G. inflata*. Therefore, it can be concluded that both the different licorice cultivars and drying heat treatment had significant effects on antioxidant activity and the production of differential metabolites. Metabolic pathway analysis revealed that metabolite changes before and after drying were predominantly related to isoflavonoid biosynthesis, metabolic pathways, secondary metabolite biosynthesis, and flavonoid biosynthesis; flavone and flavonol biosynthesis were also related. These results provide a theoretical basis for further understanding the synthesis pathways of major monomeric flavonoids in different licorice varieties. However, this study did not compare the flavonoid metabolites of dried licorice produced using different drying methods, and further research is required.

## CRediT authorship contribution statement

**Lichun Zhu:** Writing – review & editing, Supervision, Conceptualization. **Mengqin Li:** Writing – review & editing, Supervision. **Xuetao Zhang:** Writing – original draft, Resources, Formal analysis. **Qian Zhang:** Validation, Methodology, Investigation. **Xuhai Yang:** Investigation, Data curation. **Zhihua Geng:** Validation, Funding acquisition.

## Declaration of competing interest

The authors declare that they have no known competing financial interests or personal relationships that could have appeared to influence the work reported in this paper.

## Data Availability

The datasets generated for this study are available upon request from the corresponding author.

## Appendix A Supplementary data

Supplementary material Image 1
