# Supplementary material for: Metabolomics reveals the differential regulatory mechanisms of quality and flavonoid biosynthetic pathways during the drying process of varieties licorice
Source: Food Chem X. 2025 Jun 4;28:102631. doi: 10.1016/j.fochx.2025.102631 (PMC12173655; doi:10.1016/j.fochx.2025.102631)
Supplement: Supplementary file 1 — Supplementary material [file mmc1.docx]

**Metabolomics reveals the differential regulatory mechanisms of quality and flavonoid biosynthetic pathways during the drying process of varieties licorice**

Lichun Zhu^a^, Mengqin Li^a^, Xuetao Zhang^a^, Qian Zhang^a, b,^ *, Xuhai Yang^a, c^, and Zhihua Geng^a,^ *

^a^ College of Mechanical and Electrical Engineering, Shihezi University, Shihezi, China

^b^ Xinjiang Production and Construction Corps Key Laboratory of Modern Agricultural Machinery, Shihezi, China

^c^ Engineering Research Center for Production Mechanization of Oasis Special Economic Crop, Ministry of Education, Shihezi, China

*Correspondence:

Zhihua Geng; E-mail: [gzhjdxy@shzu.edu.cn](mailto:gzhjdxy@shzu.edu.cn,); Tel: 86-09932057550

Qian Zhang; E-mail: [zq_mac@shzu.edu.cn](mailto:zq_mac@shzu.edu.cn,); Tel: 86-09932057550

**Postal address:** College of Mechanical and Electrical Engineering, Xinjiang Production and Construction Corps Key Laboratory of Modern Agricultural Machinery, Shihezi University, Shihezi832000, China

The following figure shows the total ion flow diagram (TIC) and extracted ion flow diagram (XIC), with the abscess being the retention Time (Time, min) of detection, and the ordinate being the Intensity (cps) of ion detection.


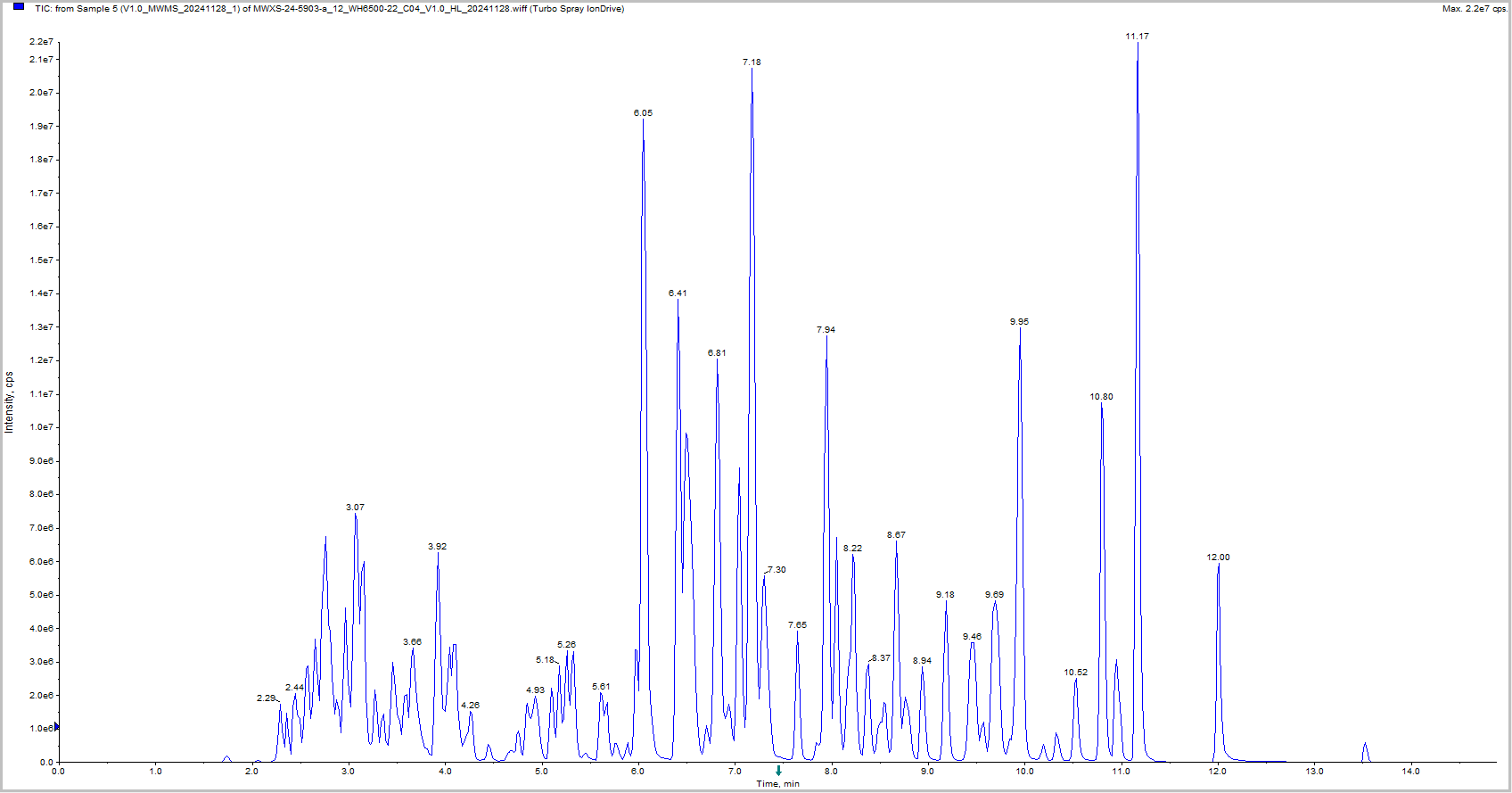


**Fig S1**: Total ion flow diagram


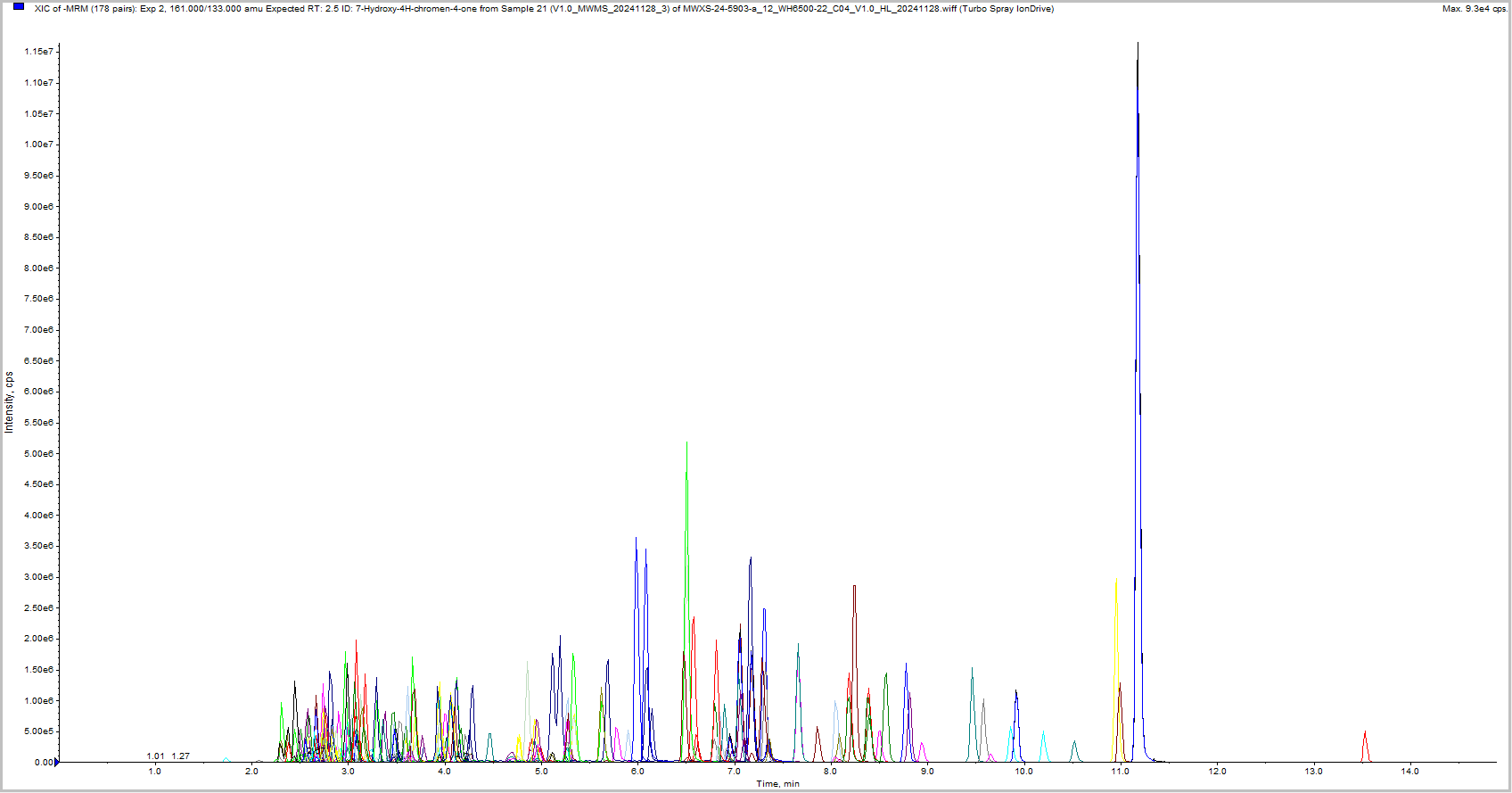


**Fig S2**: Extraction of ion flow pattern

The following figure shows the quantitative analysis and integral correction results of a random substance in different samples. The horizontal coordinate is the retention Time (min) of the detection, and the vertical coordinate is the ion current Intensity (cps) of the ion detection.


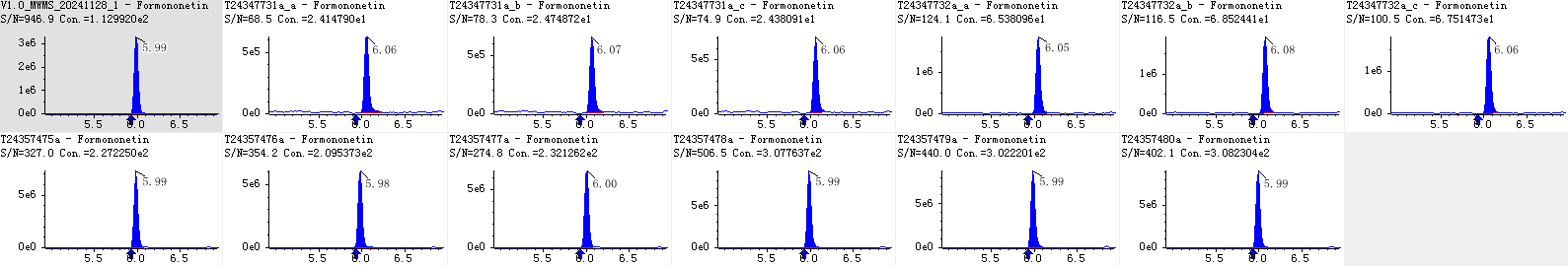


**Fig S3**: Integral correction diagram

Note: The peak Area (Area) represents the relative amount of the substance in the sample.

The following figure shows total ion flow diagram (TIC) of the mixed QC sample.


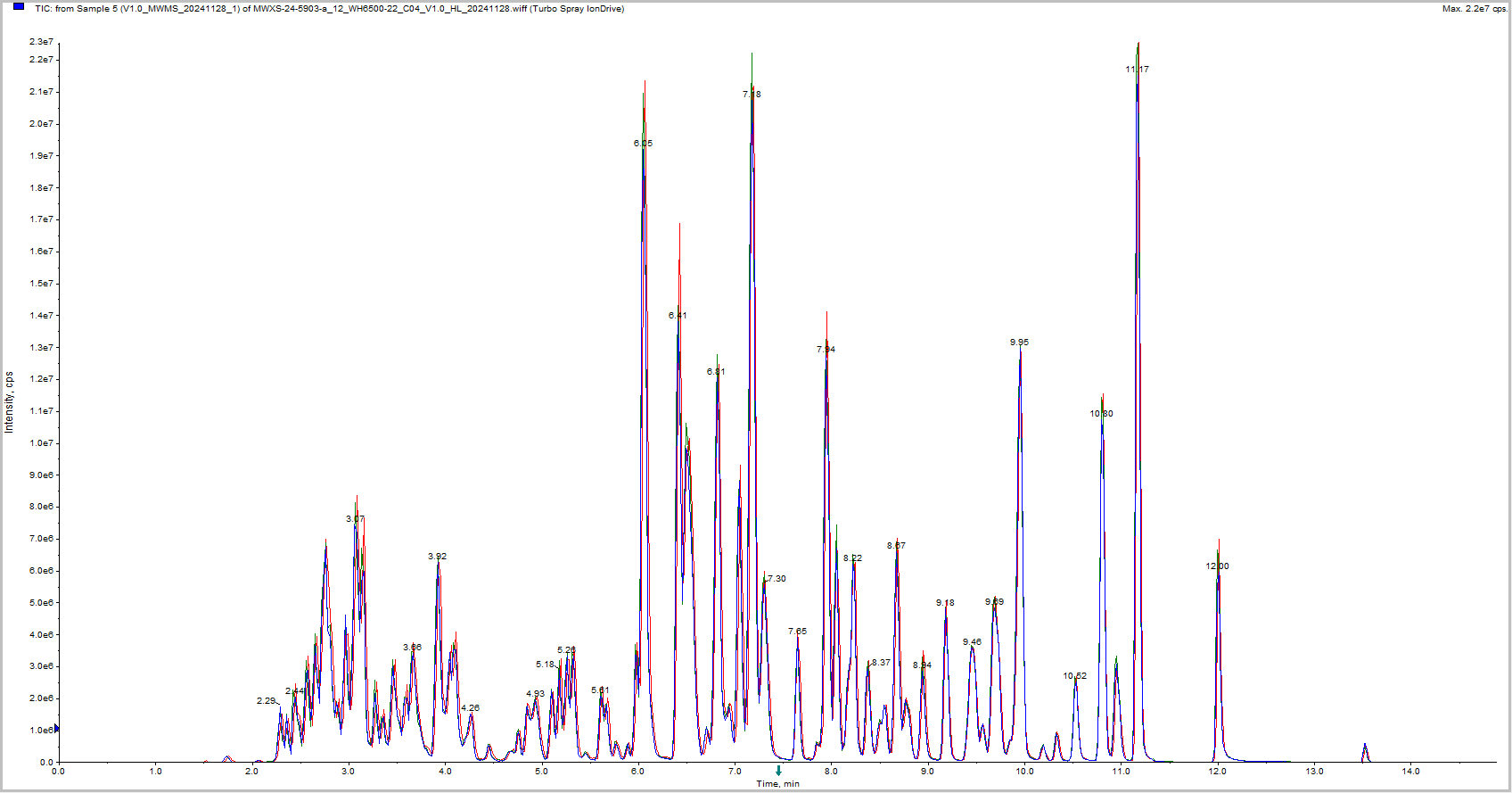


**Fig S4**: TIC overlay chart

Note: The curve overlap of the total ion flow detected by metabolites is high, that is, the retention time and peak intensity are consistent, which indicates that the signal stability is better when the same sample is detected by mass spectrometry at different times. The high stability of the instrument provides an important guarantee for the repeatability and reliability of data.

The following figure shows the coefficient of variation (CV) distribution in each group of samples.


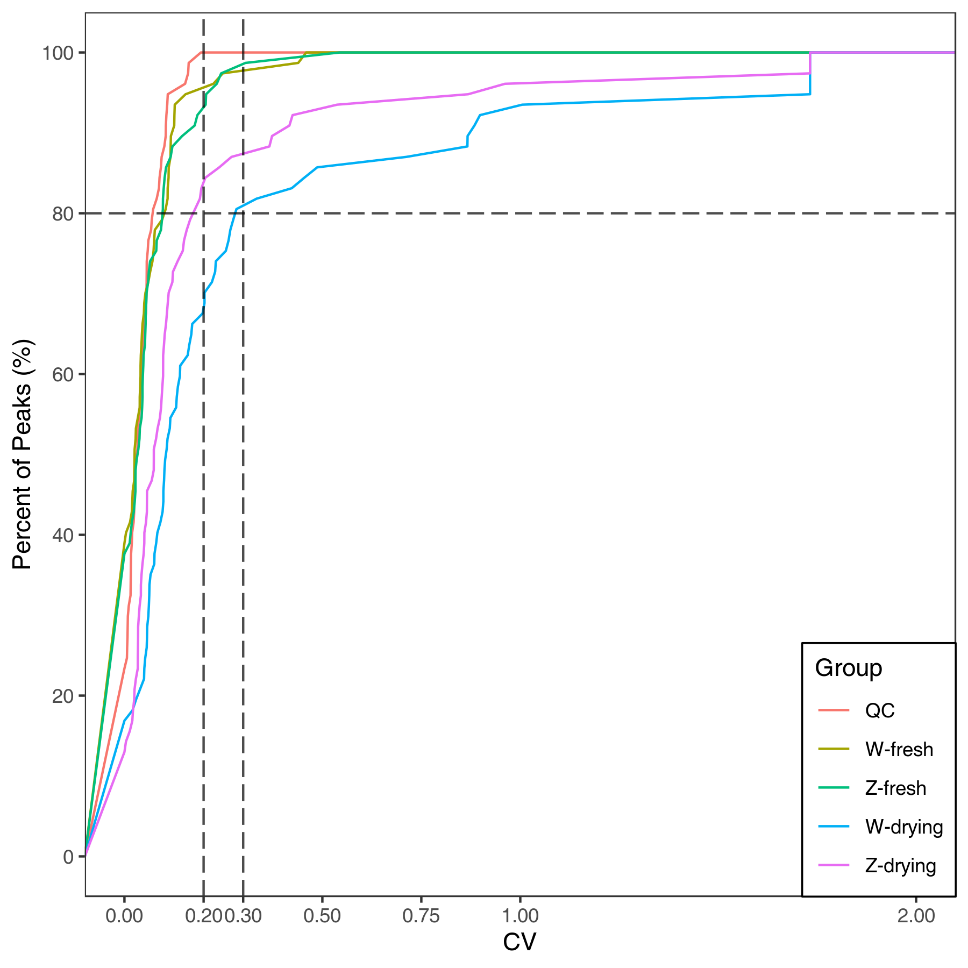


**Fig S5**: CV distribution in each group of samples

Note: The horizontal coordinate represents the CV value, the vertical coordinate represents the proportion of the number of substances less than the corresponding CV value in the total number of substances, different colors represent different grouped samples, QC is the quality control sample, in which the CV value corresponding to the two reference lines perpendicular to the X-axis is 0.2 and 0.3, and the number of substances corresponding to the reference lines parallel to the X-axis accounts for 80% of the total number of substances.

The linear equation of the standard curve and the correlation coefficient of the substances tested in this project are shown in the following table S1.

**Table S1**: Table of linear equations

| **Index** | **Class** | **RT** | **Equation** |
| --- | --- | --- | --- |
| Flavonoid_142 | - | 10.33 | y = 18192.34873 x + 14871.98611 |
| Flavonoid_133 | - | 12.00 | y = 1.38501e5 x + 9.28431e4 |
| Flavonoid_11 | - | 2.08 | y = 935.27236 x + 515.27930 |
| Flavonoid_77 | - | 3.71 | y = 22203.48981 x + 376.61226 |
| Flavonoid_94 | - | 3.80 | y = 6620.19051 x - 374.10286 |
| Flavonoid_83 | - | 3.89 | y = 26287.68887 x + 6978.63470 |
| Flavonoid_85 | - | 3.90 | y = 360.64207 x + 9111.03694 |
| Flavonoid_109 | - | 9.54 | y = 3201.74261 x + 1620.22498 |
| Flavonoid_125 | Biflavonoids | 5.63 | y = 3.28899e4 x + 19463.60504 |
| Flavonoid_47 | Biflavonoids | 8.18 | y = 3.81425e4 x + 3.37212e4 |
| Flavonoid_34 | Biflavonoids | 8.38 | y = 4.00302e4 x + 3.53304e4 |
| Flavonoid_116 | Chalcones | 11.18 | y = 2.78962e5 x + 8.22746e4 |
| Flavonoid_65 | Chalcones | 3.30 | y = 27842.32107 x + 3385.40514 |
| Flavonoid_31 | Chalcones | 3.36 | y = 18112.60425 x + 3046.45633 |
| Flavonoid_201 | Chalcones | 3.53 | y = 4.65717e4 x + 2343.66538 |
| Flavonoid_22 | Chalcones | 3.62 | y = 3.48715e4 x + 826.34653 |
| Flavonoid_177 | Chalcones | 4.86 | y = 4.22798e4 x + 8074.10880 |
| Flavonoid_112 | Chalcones | 4.89 | y = 4817.27490 x + 3869.90094 |
| Flavonoid_191 | Chalcones | 4.94 | y = 20813.19944 x + 11858.12982 |
| Flavonoid_30 | Chalcones | 5.68 | y = 4.35536e4 x + 12993.27746 |
| Flavonoid_44 | Chalcones | 6.80 | y = 23233.01095 x + 7510.16812 |
| Flavonoid_163 | Chalcones | 6.93 | y = 6813.90602 x + 2317.91079 |
| Flavonoid_134 | Chalcones | 7.36 | y = 10173.71938 x + 2926.08209 |
| Flavonoid_70 | Chalcones | 7.86 | y = 15138.85438 x + 2637.65795 |
| Flavonoid_75 | Chalcones | 8.09 | y = 11731.77027 x + 4517.00504 |
| Flavonoid_03 | Chalcones | 9.18 | y = 1.45456e5 x + 3.58913e4 |
| Flavonoid_130 | Chalcones | 9.46 | y = 3.94401e4 x + 15301.35703 |
| Flavonoid_200 | Chalcones | 9.48 | y = 7.57784e4 x + 13291.30209 |
| Flavonoid_97 | Chalcones | 9.58 | y = 28828.03042 x + 4368.62550 |
| Flavonoid_55 | Flavanols | 2.26 | y = 882.64757 x - 369.99167 |
| Flavonoid_147 | Flavanols | 2.45 | y = 1787.31365 x + 9363.62522 |
| Flavonoid_184 | Flavanols | 2.56 | y = 2730.56378 x + 7476.91068 |
| Flavonoid_150 | Flavanols | 2.85 | y = 10395.59357 x + 258.02918 |
| Flavonoid_78 | Flavanones | 2.67 | y = 23247.25999 x + 7247.20565 |
| Flavonoid_69 | Flavanones | 2.82 | y = 22066.52094 x + 4671.62914 |
| Flavonoid_48 | Flavanones | 2.90 | y = 19916.62639 x + 18611.55136 |
| Flavonoid_101 | Flavanones | 3.07 | y = 3.07709e4 x + 8010.93029 |
| Flavonoid_176 | Flavanones | 3.13 | y = 24751.51088 x + 5689.47484 |
| Flavonoid_15 | Flavanones | 3.17 | y = 3.09084e4 x + 2476.24695 |
| Flavonoid_179 | Flavanones | 4.06 | y = 3.08925e4 x + 5608.93029 |
| Flavonoid_12 | Flavanones | 4.12 | y = 3.77928e4 x + 6789.73589 |
| Flavonoid_126 | Flavanones | 4.13 | y = 2826.63284 x + 8941.54425 |
| Flavonoid_141 | Flavanones | 4.46 | y = 12909.72257 x + 337.97839 |
| Flavonoid_132 | Flavanones | 5.26 | y = 6004.98417 x + 2880.50271 |
| Flavonoid_41 | Flavanones | 5.27 | y = 24360.30418 x + 3462.84221 |
| Flavonoid_149 | Flavanones | 6.60 | y = 10666.63100 x + 4766.44617 |
| Flavonoid_158 | Flavanones | 7.10 | y = 11040.33965 x + 2126.71832 |
| Flavonoid_108 | Flavanones | 7.18 | y = 3918.83040 x + 1921.14136 |
| Flavonoid_199 | Flavanones | 7.37 | y = 560.54305 x - 878.48122 |
| Flavonoid_93 | Flavanones | 7.66 | y = 4.95220e4 x + 8688.75894 |
| Flavonoid_100 | Flavanones | 8.56 | y = 4.80322e4 x + 7188.63619 |
| Flavonoid_120 | Flavanones | 9.92 | y = 3.28963e4 x + 1804.84472 |
| Flavonoid_113 | Flavanonols | 2.62 | y = 3284.03021 x - 2113.43096 |
| Flavonoid_40 | Flavanonols | 2.64 | y = 3805.23921 x + 1173.21100 |
| Flavonoid_01 | Flavanonols | 2.92 | y = 5982.07349 x + 1471.22723 |
| Flavonoid_56 | Flavanonols | 3.09 | y = 5968.11781 x + 4294.87316 |
| Flavonoid_117 | Flavanonols | 3.19 | y = 4114.02676 x + 1271.62760 |
| Flavonoid_139 | Flavanonols | 3.59 | y = 9292.65861 x + 761.23154 |
| Flavonoid_124 | Flavanonols | 3.65 | y = 7060.66011 x + 1660.20990 |
| Flavonoid_89 | Flavanonols | 4.69 | y = 4169.90819 x - 23.47764 |
| Flavonoid_04 | Flavanonols | 4.96 | y = 9604.89038 x + 676.31984 |
| Flavonoid_51 | Flavone glycosides | 2.39 | y = 7630.87004 x + 3674.83282 |
| Flavonoid_155 | Flavone glycosides | 2.62 | y = 2039.28906 x + 738.98905 |
| Flavonoid_154 | Flavone glycosides | 2.74 | y = 28564.25159 x + 3579.60802 |
| Flavonoid_28 | Flavones | 2.78 | y = 9701.13957 x + 349.59308 |
| Flavonoid_90 | Flavones | 2.80 | y = 14116.05710 x + 1738.38080 |
| Flavonoid_42 | Flavones | 2.84 | y = 16239.75008 x - 1508.67265 |
| Flavonoid_118 | Flavones | 2.87 | y = 14217.72280 x + 573.19028 |
| Flavonoid_167 | Flavones | 2.97 | y = 3.93214e4 x - 386.61148 |
| Flavonoid_161 | Flavones | 2.99 | y = 4.05032e4 x + 5651.15379 |
| Flavonoid_160 | Flavones | 3.08 | y = 14169.36783 x + 737.50937 |
| Flavonoid_25 | Flavones | 3.10 | y = 12086.96236 x + 3067.66098 |
| Flavonoid_165 | Flavones | 3.15 | y = 25718.03209 x + 3517.03308 |
| Flavonoid_49 | Flavones | 3.48 | y = 13518.37436 x + 7603.94590 |
| Flavonoid_33 | Flavones | 3.54 | y = 18703.05780 x - 4117.65754 |
| Flavonoid_196 | Flavones | 3.67 | y = 3.47070e4 x + 8833.29410 |
| Flavonoid_190 | Flavones | 3.69 | y = 7305.22526 x + 8627.80907 |
| Flavonoid_204 | Flavones | 3.77 | y = 10963.29914 x - 1133.98113 |
| Flavonoid_81 | Flavones | 3.96 | y = 18128.13740 x + 7711.46899 |
| Flavonoid_50 | Flavones | 4.16 | y = 12244.53797 x + 5377.39455 |
| Flavonoid_156 | Flavones | 4.26 | y = 11425.74009 x + 7052.42225 |
| Flavonoid_136 | Flavones | 4.76 | y = 9371.32004 x + 5707.89831 |
| Flavonoid_72 | Flavones | 4.93 | y = 9894.20105 x + 3014.32526 |
| Flavonoid_59 | Flavones | 5.10 | y = 3077.00744 x + 5475.78003 |
| Flavonoid_06 | Flavones | 5.19 | y = 4.73367e4 x + 13552.82966 |
| Flavonoid_143 | Flavones | 5.34 | y = 18214.88482 x + 4351.66881 |
| Flavonoid_29 | Flavones | 5.63 | y = 26084.92163 x + 2161.65099 |
| Flavonoid_10 | Flavones | 5.78 | y = 17303.83881 x - 1414.03553 |
| Flavonoid_171 | Flavones | 5.90 | y = 13856.79115 x + 5934.11707 |
| Flavonoid_168 | Flavones | 6.05 | y = 5.11855e5 x + 1.24344e5 |
| Flavonoid_193 | Flavones | 6.08 | y = 18285.37449 x + 3933.22392 |
| Flavonoid_151 | Flavones | 6.41 | y = 3.28784e5 x + 9.21470e4 |
| Flavonoid_164 | Flavones | 6.52 | y = 8.29935e4 x + 20352.97509 |
| Flavonoid_46 | Flavones | 6.57 | y = 6.31408e4 x + 11672.53779 |
| Flavonoid_198 | Flavones | 6.80 | y = 4.64408e4 x + 14132.99624 |
| Flavonoid_39 | Flavones | 6.81 | y = 9.91906e4 x + 3.09721e4 |
| Flavonoid_140 | Flavones | 6.85 | y = 20027.87325 x + 5000.03495 |
| Flavonoid_24 | Flavones | 6.90 | y = 23598.11112 x + 12288.75800 |
| Flavonoid_107 | Flavones | 6.96 | y = 1926.21279 x + 2186.46323 |
| Flavonoid_61 | Flavones | 6.98 | y = 5795.32926 x + 2081.68015 |
| Flavonoid_76 | Flavones | 7.05 | y = 3.69488e4 x + 10614.14241 |
| Flavonoid_99 | Flavones | 7.06 | y = 5.10903e4 x + 10983.75597 |
| Flavonoid_82 | Flavones | 7.07 | y = 3.18611e4 x + 6213.33366 |
| Flavonoid_14 | Flavones | 7.16 | y = 1.26308e5 x + 7.21323e4 |
| Flavonoid_166 | Flavones | 7.18 | y = 4.62202e4 x + 9052.07773 |
| Flavonoid_103 | Flavones | 7.19 | y = 3.53777e5 x + 7.19177e4 |
| Flavonoid_71 | Flavones | 7.25 | y = 1404.18228 x + 827.45172 |
| Flavonoid_21 | Flavones | 7.94 | y = 4.58186e5 x + 6.29229e4 |
| Flavonoid_173 | Flavones | 8.67 | y = 1.24873e5 x + 26675.63247 |
| Flavonoid_88 | Flavones | 8.68 | y = 8.65818e4 x + 25005.07948 |
| Flavonoid_128 | Flavones | 9.42 | y = 3.87206e4 x + 11306.22844 |
| Flavonoid_162 | Flavones | 9.68 | y = 1.25230e5 x + 4.69500e4 |
| Flavonoid_16 | Flavones | 9.72 | y = 7.93441e4 x + 3.20669e4 |
| Flavonoid_159 | Flavonols | 10.52 | y = 10434.77396 x + 4059.44981 |
| Flavonoid_189 | Flavonols | 2.45 | y = 3.36374e4 x + 5599.76573 |
| Flavonoid_138 | Flavonols | 2.46 | y = 14618.40867 x - 3780.49670 |
| Flavonoid_106 | Flavonols | 2.56 | y = 8185.92725 x + 960.89747 |
| Flavonoid_169 | Flavonols | 2.65 | y = 11975.94451 x - 1774.97073 |
| Flavonoid_57 | Flavonols | 2.67 | y = 21363.15563 x + 3061.93791 |
| Flavonoid_52 | Flavonols | 2.72 | y = 7893.98848 x + 3206.02435 |
| Flavonoid_172 | Flavonols | 2.73 | y = 11507.67919 x + 3950.72084 |
| Flavonoid_54 | Flavonols | 2.75 | y = 23821.30313 x + 3722.57124 |
| Flavonoid_178 | Flavonols | 2.76 | y = 705.93902 x - 1877.94639 |
| Flavonoid_98 | Flavonols | 2.76 | y = 4.41304e4 x - 7632.93743 |
| Flavonoid_194 | Flavonols | 2.76 | y = 807.45654 x - 240.52596 |
| Flavonoid_123 | Flavonols | 2.77 | y = 26756.14533 x - 9.84765e4 |
| Flavonoid_02 | Flavonols | 2.78 | y = 13003.04178 x + 1651.47146 |
| Flavonoid_86 | Flavonols | 2.99 | y = 21388.70239 x - 1596.61130 |
| Flavonoid_119 | Flavonols | 3.00 | y = 15923.75201 x + 10.46927 |
| Flavonoid_197 | Flavonols | 3.04 | y = 19126.12609 x + 2902.22053 |
| Flavonoid_115 | Flavonols | 3.05 | y = 9398.25763 x - 4239.25083 |
| Flavonoid_180 | Flavonols | 3.09 | y = 4.90273e4 x + 5665.29124 |
| Flavonoid_175 | Flavonols | 3.38 | y = 23062.66529 x + 8005.11715 |
| Flavonoid_137 | Flavonols | 3.94 | y = 3.35555e4 x + 11770.14729 |
| Flavonoid_146 | Flavonols | 4.10 | y = 22920.42536 x + 5781.03491 |
| Flavonoid_45 | Flavonols | 4.15 | y = 89.71612 x + 7973.66749 |
| Flavonoid_23 | Flavonols | 4.22 | y = 6305.72852 x - 2540.90177 |
| Flavonoid_43 | Flavonols | 5.09 | y = 1018.21427 x + 1115.20715 |
| Flavonoid_58 | Flavonols | 5.28 | y = 18707.75243 x - 2916.45996 |
| Flavonoid_20 | Flavonols | 6.14 | y = 22347.99592 x + 5443.75319 |
| Flavonoid_36 | Flavonols | 6.48 | y = 4.16293e4 x + 17583.70636 |
| Flavonoid_185 | Flavonols | 6.50 | y = 1.22744e5 x + 3.35785e4 |
| Flavonoid_148 | Flavonols | 7.16 | y = 9.55900e4 x + 11158.46799 |
| Flavonoid_110 | Flavonols | 7.31 | y = 6.65862e4 x + 19633.87506 |
| Flavonoid_27 | Flavonols | 8.39 | y = 2329.28168 x + 2462.78276 |
| Flavonoid_170 | Flavonols | 8.94 | y = 8101.07812 x + 1316.29207 |
| Flavonoid_87 | Flavonols | 9.66 | y = 20731.08696 x + 9128.07336 |
| Flavonoid_38 | Isoflavanones | 10.20 | y = 14323.33258 x + 2976.97095 |
| Flavonoid_66 | Isoflavanones | 2.32 | y = 21949.53513 x + 7975.13396 |
| Flavonoid_32 | Isoflavanones | 2.57 | y = 6006.00356 x + 2939.04918 |
| Flavonoid_153 | Isoflavanones | 2.60 | y = 11482.34533 x + 1605.74683 |
| Flavonoid_96 | Isoflavanones | 2.76 | y = 11706.40431 x + 223.64317 |
| Flavonoid_73 | Isoflavanones | 2.92 | y = 3581.53861 x + 217.42051 |
| Flavonoid_114 | Isoflavanones | 3.24 | y = 5844.88731 x + 2247.48774 |
| Flavonoid_181 | Isoflavanones | 3.42 | y = 3470.14090 x + 910.67168 |
| Flavonoid_182 | Isoflavanones | 3.48 | y = 18451.92286 x + 7907.98311 |
| Flavonoid_152 | Isoflavanones | 3.59 | y = 16435.47415 x + 5480.05484 |
| Flavonoid_127 | Isoflavanones | 3.96 | y = 0.02706 x + 0.00853 |
| Flavonoid_183 | Isoflavanones | 4.01 | y = 22226.50824 x + 10534.67937 |
| Flavonoid_95 | Isoflavanones | 4.28 | y = 3.15028e4 x + 7858.97374 |
| Flavonoid_09 | Isoflavanones | 4.99 | y = 6301.00831 x + 6096.02285 |
| Flavonoid_63 | Isoflavanones | 5.12 | y = 3622.77080 x + 1181.08574 |
| Flavonoid_26 | Isoflavanones | 5.99 | y = 9.44482e4 x + 21639.64458 |
| Flavonoid_111 | Isoflavanones | 6.82 | y = 1.68068e5 x + 3.86781e4 |
| Flavonoid_131 | Isoflavanones | 7.32 | y = 4.08046e4 x + 7291.37572 |
| Flavonoid_186 | Isoflavanones | 7.35 | y = 10972.14729 x + 3007.11178 |
| Flavonoid_121 | Isoflavanones | 8.05 | y = 3.11524e4 x + 5602.59874 |
| Flavonoid_05 | Isoflavanones | 8.23 | y = 1.20961e5 x + 22453.00245 |
| Flavonoid_104 | Isoflavanones | 8.39 | y = 19758.40580 x + 5615.87834 |
| Flavonoid_174 | Isoflavanones | 8.81 | y = 28149.66815 x + 7414.56103 |
| Flavonoid_08 | Isoflavanones | 9.95 | y = 4.80135e5 x + 1.17025e5 |
| Flavonoid_60 | Other flavonoids | 10.95 | y = 7.80654e4 x + 26031.54596 |
| Flavonoid_91 | Other flavonoids | 10.99 | y = 3.27160e4 x + 10005.80526 |
| Flavonoid_37 | Other flavonoids | 7.29 | y = 5.92545e4 x + 16795.01386 |
| Flavonoid_129 | Other flavonoids | 8.77 | y = 4.86499e4 x + 4767.00599 |
| Flavonoid_105 | Phenonic acids | 10.80 | y = 3.50402e5 x + 8.25323e4 |
| Flavonoid_122 | Phenonic acids | 2.68 | y = 2197.12914 x + 4500.80316 |
| Flavonoid_79 | Phenonic acids | 2.74 | y = 10768.44413 x + 2193.20322 |
| Flavonoid_35 | Phenonic acids | 3.13 | y = 497.88729 x + 6720.66213 |
| Flavonoid_144 | Phenonic acids | 6.71 | y = 3.34472e4 x + 12141.90165 |
| Flavonoid_17 | Phenonic acids | 8.24 | y = 7.87393e4 x + 20450.92416 |
| Flavonoid_53 | Phenonic acids | 8.50 | y = 15523.32317 x + 5109.19385 |
| Flavonoid_62 | Phenonic acids | 9.86 | y = 15429.98954 x + 986.48562 |
| Flavonoid_68 | Xanthones | 2.31 | y = 7806.84692 x + 3580.68064 |
| Flavonoid_157 | Xanthones | 2.37 | y = 14225.94712 x + 3928.71804 |

The substances detected results of this project are shown in the table S2 below.

**Table S2**: Substances detected results of this project.

| **Index** | **Compounds** | **Class** | **Ion mode** | **Ionization model** | **Formula** |
| --- | --- | --- | --- | --- | --- |
| Flavonoid_117 | Engeletin | Flavanonols | Negative | [M-H]- | C21H22O10 |
| Flavonoid_183 | 2'-Hydroxygenistein | Isoflavanones | Negative | [M-H]- | C15H10O6 |
| Flavonoid_69 | Liquiritin | Flavanones | Negative | [M-H]- | C21H22O9 |
| Flavonoid_130 | Isobavachalcone | Chalcones | Negative | [M-H]- | C20H20O4 |
| Flavonoid_100 | Sophoraflavanone G | Flavanones | Negative | [M-H]- | C25H28O6 |
| Flavonoid_186 | Prunetin | Isoflavanones | Negative | [M-H]- | C16H12O5 |
| Flavonoid_112 | Echinatin | Chalcones | Negative | [M-H]- | C16H14O4 |
| Flavonoid_57 | Rutin | Flavonols | Negative | [M-H]- | C27H30O16 |
| Flavonoid_191 | Naringenin chalcone | Chalcones | Negative | [M-H]- | C15H12O5 |
| Flavonoid_120 | Bavachinin | Flavanones | Negative | [M-H]- | C21H22O4 |
| Flavonoid_26 | Formononetin | Isoflavanones | Negative | [M-H]- | C16H12O4 |
| Flavonoid_32 | Daidzin | Isoflavanones | Negative | [M-H]- | C21H20O9 |
| Flavonoid_66 | Puerarin | Isoflavanones | Negative | [M-H]- | C21H20O9 |
| Flavonoid_09 | Genistein | Isoflavanones | Negative | [M-H]- | C15H10O5 |
| Flavonoid_152 | Ononin | Isoflavanones | Negative | [M-H]- | C22H22O9 |
| Flavonoid_96 | Calycosin-7-O-β-D-glucoside | Isoflavanones | Negative | [M-H]- | C22H22O10 |
| Flavonoid_121 | Licoisoflavone A | Isoflavanones | Negative | [M-H]- | C20H18O6 |
| Flavonoid_139 | Dihydrokaempferol | Flavanonols | Negative | [M-H]- | C15H12O6 |
| Flavonoid_190 | 7,4'-Dihydroxyflavone | Flavones | Negative | [M-H]- | C15H10O4 |
| Flavonoid_153 | Glycitin | Isoflavanones | Negative | [M-H]- | C22H22O10 |
| Flavonoid_30 | Isoliquiritigenin | Chalcones | Negative | [M-H]- | C15H12O4 |
| Flavonoid_11 | Hydroxysafflor yellow A | - | Negative | [M-H]- | C27H32O16 |
| Flavonoid_104 | Corylin | Isoflavanones | Negative | [M-H]- | C20H16O4 |
| Flavonoid_01 | Astilbin | Flavanonols | Negative | [M-H]- | C21H22O11 |
| Flavonoid_154 | Vitexin | Flavone glycosides | Negative | [M-H]- | C21H20O10 |
| Flavonoid_170 | Licoflavonol | Flavonols | Negative | [M-H]- | C20H18O6 |
| Flavonoid_140 | Licoflavone A | Flavones | Negative | [M-H]- | C20H18O4 |
| Flavonoid_176 | Naringenin-7-glucoside | Flavanones | Negative | [M-H]- | C21H22O10 |
| Flavonoid_90 | Cynaroside | Flavones | Negative | [M-H]- | C21H20O11 |
| Flavonoid_162 | Tectochrysin | Flavones | Positive | [M+H]+ | C16H12O4 |
| Flavonoid_72 | Apigenin | Flavones | Negative | [M-H]- | C15H10O5 |
| Flavonoid_91 | Morusin | Other flavonoids | Negative | [M-H]- | C25H24O6 |
| Flavonoid_160 | Apigenin 7-glucoside | Flavones | Negative | [M-H]- | C21H20O10 |
| Flavonoid_76 | Sakuranetin | Flavones | Negative | [M-H]- | C16H14O5 |
| Flavonoid_97 | Xanthohumol | Chalcones | Negative | [M-H]- | C21H22O5 |
| Flavonoid_167 | Oroxin B | Flavones | Negative | [M-H]- | C27H30O15 |
| Flavonoid_93 | Bavachin | Flavanones | Negative | [M-H]- | C20H20O4 |
| Flavonoid_79 | Ligustroflavone | Phenonic acids | Negative | [M-H]- | C33H40O18 |
| Flavonoid_189 | Robinin | Flavonols | Negative | [M-H]- | C33H40O19 |
| Flavonoid_51 | 3'-Methoxypuerarin | Flavone glycosides | Negative | [M-H]- | C22H22O10 |
| Flavonoid_182 | 2'-Hydroxydaidzein | Isoflavanones | Negative | [M-H]- | C15H10O5 |
| Flavonoid_119 | Astragalin | Flavonols | Negative | [M-H]- | C21H20O11 |
| Flavonoid_73 | Genistin | Isoflavanones | Negative | [M-H]- | C21H20O10 |
| Flavonoid_108 | Pinocembrin | Flavanones | Negative | [M-H]- | C15H12O4 |
| Flavonoid_50 | Luteolin | Flavones | Negative | [M-H]- | C15H10O6 |
| Flavonoid_118 | Narcissin | Flavones | Negative | [M-H]- | C28H32O16 |
| Flavonoid_133 | β-Anhydroicaritin | - | Positive | [M+H]+ | C21H20O6 |
| Flavonoid_43 | Kaempferol | Flavonols | Negative | [M-H]- | C15H10O6 |
| Flavonoid_23 | Quercetin | Flavonols | Negative | [M-H]- | C15H10O7 |
| Flavonoid_127 | Daidzein | Isoflavanones | Negative | [M-H]- | C15H10O4 |
| Flavonoid_12 | Liquiritigenin | Flavanones | Negative | [M-H]- | C15H12O4 |
| Flavonoid_147 | (-)-Epicatechin | Flavanols | Negative | [M-H]- | C15H14O6 |
| Flavonoid_172 | Kaempferitrin | Flavonols | Negative | [M-H]- | C27H30O14 |
| Flavonoid_131 | Neobavaisoflavone | Isoflavanones | Negative | [M-H]- | C20H18O4 |
| Flavonoid_138 | Baimaside | Flavonols | Negative | [M-H]- | C27H30O17 |
| Flavonoid_126 | Eriodictyol | Flavanones | Negative | [M-H]- | C15H12O6 |
| Flavonoid_95 | Calycosin | Isoflavanones | Negative | [M-H]- | C16H12O5 |
| Flavonoid_58 | Isorhamnetin | Flavonols | Negative | [M-H]- | C16H12O7 |
| Flavonoid_155 | Spinosin | Flavone glycosides | Negative | [M-H]- | C28H32O15 |
| Flavonoid_194 | Quercimeritrin | Flavonols | Negative | [M-H]- | C21H20O12 |
| Flavonoid_158 | Isosakuranetin | Flavanones | Negative | [M-H]- | C16H14O5 |
| Flavonoid_56 | Taxifolin | Flavanonols | Negative | [M-H]- | C15H12O7 |
| Flavonoid_137 | Tiliroside | Flavonols | Negative | [M-H]- | C30H26O13 |
| Flavonoid_166 | Genkwanin | Flavones | Negative | [M-H]- | C16H12O5 |
| Flavonoid_185 | 3,7-Di-O-methylquercetin | Flavonols | Negative | [M-H]- | C17H14O7 |
| Flavonoid_06 | Diosmetin | Flavones | Negative | [M-H]- | C16H12O6 |
| Flavonoid_174 | Glabridin | Isoflavanones | Negative | [M-H]- | C20H20O4 |
| Flavonoid_110 | Kaempferide | Flavonols | Negative | [M-H]- | C16H12O6 |
| Flavonoid_75 | Licochalcone C | Chalcones | Negative | [M-H]- | C21H22O4 |
| Flavonoid_41 | Hesperetin | Flavanones | Negative | [M-H]- | C16H14O6 |
| Flavonoid_122 | 7-Hydroxy-4H-chromen-4-one | Phenonic acids | Negative | [M-H]- | C9H6O3 |
| Flavonoid_82 | Chrysosplenetin | Flavones | Negative | [M-H]- | C19H18O8 |
| Flavonoid_42 | Nicotiflorin | Flavones | Negative | [M-H]- | C27H30O15 |
| Flavonoid_21 | Tangeretin | Flavones | Positive | [M+H]+ | C20H20O7 |
| Flavonoid_68 | Mangiferin | Xanthones | Negative | [M-H]- | C19H18O11 |
| Flavonoid_98 | Hyperoside | Flavonols | Positive | [M+H]+ | C21H20O12 |
| Flavonoid_113 | Taxifolin 7-O-rhamnoside | Flavanonols | Negative | [M-H]- | C21H22O11 |
| Flavonoid_35 | Syringaldehyde | Phenonic acids | Negative | [M-H]- | C9H10O4 |
| Flavonoid_36 | Icariside I | Flavonols | Negative | [M-H]- | C27H30O11 |
| Flavonoid_62 | Methylophiopogonanone B | Phenonic acids | Negative | [M-H]- | C19H20O5 |
| Flavonoid_175 | Afzelin | Flavonols | Negative | [M-H]- | C21H20O10 |
| Flavonoid_27 | Flavonol | Flavonols | Positive | [M+H]+ | C15H10O3 |
| Flavonoid_115 | Quercitrin | Flavonols | Negative | [M-H]- | C21H20O11 |
| Flavonoid_144 | Kavain | Phenonic acids | Positive | [M+H]+ | C14H14O3 |
| Flavonoid_17 | Flavokawain C | Phenonic acids | Negative | [M-H]- | C17H16O5 |
| Flavonoid_53 | (E)-Cardamonin | Phenonic acids | Negative | [M-H]- | C16H14O4 |
| Flavonoid_169 | Quercetin 3-O-(6''-galloyl)-β-D-galactopyranoside | Flavonols | Negative | [M-H]- | C28H24O16 |
| Flavonoid_20 | Sagittatoside A | Flavonols | Negative | [M-H]- | C33H40O15 |
| Flavonoid_52 | Kaempferol 3-neohesperidoside | Flavonols | Negative | [M-H]- | C27H30O15 |
| Flavonoid_197 | Isorhamnetin 3-O-glucoside | Flavonols | Negative | [M-H]- | C22H22O12 |
| Flavonoid_63 | Tectorigenin | Isoflavanones | Negative | [M-H]- | C16H12O6 |
| Flavonoid_105 | (E)-Flavokawain A | Phenonic acids | Positive | [M+H]+ | C18H18O5 |
| Flavonoid_180 | Spiraeoside | Flavonols | Negative | [M-H]- | C21H20O12 |
| Flavonoid_148 | Baohuoside I | Flavonols | Negative | [M-H]- | C27H30O10 |
| Flavonoid_02 | Miquelianin | Flavonols | Negative | [M-H]- | C21H18O13 |
| Flavonoid_38 | Corylifol A | Isoflavanones | Negative | [M-H]- | C25H26O4 |
| Flavonoid_159 | Icaritin | Flavonols | Negative | [M-H]- | C21H20O6 |
| Flavonoid_54 | Isorhamnetin-3-O-neohespeidoside | Flavonols | Negative | [M-H]- | C28H32O16 |
| Flavonoid_146 | Icariin | Flavonols | Negative | [M-H]- | C33H40O15 |
| Flavonoid_111 | Irisflorentin | Isoflavanones | Positive | [M+H]+ | C20H18O8 |
| Flavonoid_114 | 6''-O-Acetylglycitin | Isoflavanones | Negative | [M-H]- | C24H24O11 |
| Flavonoid_178 | Myricitrin | Flavonols | Negative | [M-H]- | C21H20O12 |
| Flavonoid_08 | 5-Methyl-7-methoxyisoflavone | Isoflavanones | Positive | [M+H]+ | C17H14O3 |
| Flavonoid_181 | Demethyltexasin | Isoflavanones | Negative | [M-H]- | C15H10O5 |
| Flavonoid_106 | Typhaneoside | Flavonols | Negative | [M-H]- | C34H42O20 |
| Flavonoid_37 | Kurarinone | Other flavonoids | Negative | [M-H]- | C26H30O6 |
| Flavonoid_60 | Kushenol A | Other flavonoids | Negative | [M-H]- | C25H28O5 |
| Flavonoid_123 | 2''-O-Galloylhyperin | Flavonols | Negative | [M-H]- | C28H24O16 |
| Flavonoid_87 | Noricaritin | Flavonols | Positive | [M+H]+ | C20H20O7 |
| Flavonoid_129 | Mulberrin | Other flavonoids | Negative | [M-H]- | C25H26O6 |
| Flavonoid_86 | Avicularin | Flavonols | Negative | [M-H]- | C20H18O11 |
| Flavonoid_05 | 7-Methoxyisoflavone | Isoflavanones | Positive | [M+H]+ | C16H12O3 |
| Flavonoid_94 | Epimedin B | - | Positive | [M+H]+ | C38H48O19 |
| Flavonoid_39 | Trimethylapigenin | Flavones | Positive | [M+H]+ | C18H16O5 |
| Flavonoid_165 | Homoplantaginin | Flavones | Negative | [M-H]- | C22H22O11 |
| Flavonoid_44 | 4-Hydroxychalcone | Chalcones | Negative | [M-H]- | C15H12O2 |
| Flavonoid_201 | Trilobatin | Chalcones | Negative | [M-H]- | C21H24O10 |
| Flavonoid_150 | (-)-Catechin gallate | Flavanols | Negative | [M-H]- | C22H18O10 |
| Flavonoid_184 | Afzelechin | Flavanols | Negative | [M-H]- | C15H14O5 |
| Flavonoid_55 | (-)-Catechin | Flavanols | Negative | [M-H]- | C15H14O6 |
| Flavonoid_48 | Narirutin | Flavanones | Negative | [M-H]- | C27H32O14 |
| Flavonoid_31 | Naringin Dihydrochalcone | Chalcones | Negative | [M-H]- | C27H34O14 |
| Flavonoid_78 | Eriocitrin | Flavanones | Negative | [M-H]- | C27H32O15 |
| Flavonoid_15 | Neohesperidin | Flavanones | Negative | [M-H]- | C28H34O15 |
| Flavonoid_132 | Alpinetin | Flavanones | Negative | [M-H]- | C16H14O4 |
| Flavonoid_141 | Isosakuranin | Flavanones | Negative | [M-H]- | C22H24O10 |
| Flavonoid_149 | Farrerol | Flavanones | Negative | [M-H]- | C17H16O5 |
| Flavonoid_179 | Poncirin | Flavanones | Negative | [M-H]- | C28H34O14 |
| Flavonoid_199 | Persicogenin | Flavanones | Negative | [M-H]- | C17H16O6 |
| Flavonoid_101 | Hesperidin | Flavanones | Negative | [M-H]- | C28H34O15 |
| Flavonoid_40 | Dihydromyricetin | Flavanonols | Negative | [M-H]- | C15H12O8 |
| Flavonoid_65 | Phlorizin | Chalcones | Negative | [M-H]- | C21H24O10 |
| Flavonoid_22 | Neohesperidin dihydrochalcone | Chalcones | Negative | [M-H]- | C28H36O15 |
| Flavonoid_142 | Sciadopitysin | - | Positive | [M+H]+ | C33H24O10 |
| Flavonoid_77 | Epimedin A | - | Positive | [M+H]+ | C39H50O20 |
| Flavonoid_109 | Deguelin | - | Positive | [M+H]+ | C23H22O6 |
| Flavonoid_83 | Epmedin C | - | Positive | [M+H]+ | C39H50O19 |
| Flavonoid_85 | Methylnissolin-3-O-glucoside | - | Positive | [M+H]+ | C23H26O10 |
| Flavonoid_125 | Amentoflavone | Biflavonoids | Negative | [M-H]- | C30H18O10 |
| Flavonoid_70 | Licochalcone E | Chalcones | Negative | [M-H]- | C21H22O4 |
| Flavonoid_47 | Ginkgetin | Biflavonoids | Negative | [M-H]- | C32H22O10 |
| Flavonoid_03 | 4,4'-Dimethoxychalcone | Chalcones | Positive | [M+H]+ | C17H16O3 |
| Flavonoid_134 | Loureirin B | Chalcones | Negative | [M-H]- | C18H20O5 |
| Flavonoid_200 | Benzylideneacetophenone | Chalcones | Positive | [M+H]+ | C15H12O |
| Flavonoid_163 | 4'-Hydroxychalcone | Chalcones | Negative | [M-H]- | C15H12O2 |
| Flavonoid_177 | Phloretin | Chalcones | Negative | [M-H]- | C15H14O5 |
| Flavonoid_116 | 4'-O-Methylbavachalcone | Chalcones | Negative | [M-H]- | C21H22O4 |
| Flavonoid_34 | Isoginkgetin | Biflavonoids | Negative | [M-H]- | C32H22O10 |
| Flavonoid_45 | 3,7,4'-Trihydroxyflavone | Flavonols | Positive | [M+H]+ | C15H10O5 |
| Flavonoid_124 | Silychristin | Flavanonols | Negative | [M-H]- | C25H22O10 |
| Flavonoid_04 | Isosilybin | Flavanonols | Negative | [M-H]- | C25H22O10 |
| Flavonoid_59 | Hispidulin | Flavones | Negative | [M-H]- | C16H12O6 |
| Flavonoid_49 | Baicalin | Flavones | Negative | [M-H]- | C21H18O11 |
| Flavonoid_46 | Eupatorin | Flavones | Negative | [M-H]- | C18H16O7 |
| Flavonoid_33 | Oroxin A | Flavones | Negative | [M-H]- | C21H20O10 |
| Flavonoid_29 | IKarisoside A | Flavones | Negative | [M-H]- | C26H28O10 |
| Flavonoid_25 | Apigenin-7-glucuronide | Flavones | Negative | [M-H]- | C21H18O11 |
| Flavonoid_61 | Chrysin | Flavones | Negative | [M-H]- | C15H10O4 |
| Flavonoid_24 | 4',5-Dihydroxyflavone | Flavones | Negative | [M-H]- | C15H10O4 |
| Flavonoid_99 | Acacetin | Flavones | Negative | [M-H]- | C16H12O5 |
| Flavonoid_28 | Scutellarin | Flavones | Negative | [M-H]- | C21H18O12 |
| Flavonoid_107 | Wogonin | Flavones | Negative | [M-H]- | C16H12O5 |
| Flavonoid_10 | 3,4'-Dihydroxyflavone | Flavones | Negative | [M-H]- | C15H10O4 |
| Flavonoid_103 | Scutellarein tetramethyl ether | Flavones | Positive | [M+H]+ | C19H18O6 |
| Flavonoid_161 | Diosmin | Flavones | Negative | [M-H]- | C28H32O15 |
| Flavonoid_128 | 5-Hydroxyflavone | Flavones | Positive | [M+H]+ | C15H10O3 |
| Flavonoid_89 | Silibinin | Flavanonols | Negative | [M-H]- | C25H22O10 |
| Flavonoid_71 | Galangin | Flavones | Negative | [M-H]- | C15H10O5 |
| Flavonoid_88 | 5-O-Demethylnobiletin | Flavones | Positive | [M+H]+ | C20H20O8 |
| Flavonoid_196 | Pedalitin | Flavones | Negative | [M-H]- | C16H12O7 |
| Flavonoid_193 | hydroxygenkwanin | Flavones | Positive | [M+H]+ | C16H12O6 |
| Flavonoid_173 | 6-Methylflavone | Flavones | Positive | [M+H]+ | C16H12O2 |
| Flavonoid_171 | 6-Hydroxyflavone | Flavones | Negative | [M-H]- | C15H10O3 |
| Flavonoid_151 | Sinensetin | Flavones | Positive | [M+H]+ | C20H20O7 |
| Flavonoid_198 | 5,7-Dihydroxy-3',4',5'-trimethoxyflavone | Flavones | Positive | [M+H]+ | C18H16O7 |
| Flavonoid_81 | Tricin | Flavones | Negative | [M-H]- | C17H14O7 |
| Flavonoid_16 | 7,4'-Di-O-methylapigenin | Flavones | Positive | [M+H]+ | C17H14O5 |
| Flavonoid_143 | Jaceosidin | Flavones | Negative | [M-H]- | C17H14O7 |
| Flavonoid_14 | Nobiletin | Flavones | Positive | [M+H]+ | C21H22O8 |
| Flavonoid_136 | 6,2'-Dihydroxyflavone | Flavones | Negative | [M-H]- | C15H10O4 |
| Flavonoid_168 | 5,7,3',4'-Tetramethoxyflavone | Flavones | Positive | [M+H]+ | C19H18O6 |
| Flavonoid_164 | 5-Methoxyflavone | Flavones | Positive | [M+H]+ | C16H12O3 |
| Flavonoid_204 | Linarin | Flavones | Negative | [M-H]- | C28H32O14 |
| Flavonoid_156 | Wogonoside | Flavones | Negative | [M-H]- | C22H20O11 |
| Flavonoid_157 | Isomangiferin | Xanthones | Negative | [M-H]- | C19H18O11 |

Note: Each column represents a specific meaning. Index: short for substance; Compounds: English names of substances; Class: material English category; Ion mode: ion mode; Ionization model: ionization mode (M+H is positively charged, M-H is negatively charged); Formula: The molecular formula of a substance.
